# Extracellular vesicle phenotypic patterns associated with CMV, EBV and HHV-6 reactivation after haploidentical transplantation with post-transplant cyclophosphamide

**DOI:** 10.3389/fimmu.2026.1937675

**Published:** 2026-10-05

**Authors:** Giuseppe Lia, Clara Di Vito, Stefania Bruno, Jacopo Mariotti, Alessia Melis, Michela Calvi, Luisa Giaccone, Armando Santoro, Stefania Bramanti, Luca Castagna, Andrea Evangelista, Domenico Mavilio, Benedetto Bruno

**Affiliations:** 1 Division of Hematology, Department of Oncology, A.O.U. Città della Salute e della Scienza di Torino, Torino, Italy; 2 Department of Molecular Biotechnology and Health Sciences, University of Torino, Torino, Italy; 3 Department of Medical Biotechnologies and Translational Medicine (BioMeTra), University of Milan, Milan, Italy; 4 Unit of Clinical and Experimental Immunology, Istituti di Ricovero e Cura a Carattere Scientifico (IRCCS) Humanitas Research Hospital, Rozzano, Milan, Italy; 5 Department of Medical Sciences, University of Torino, Torino, Italy; 6 Bone Marrow Transplant Unit, IRCCS Humanitas Research Hospital, Milan, Italy; 7 Clinical Epidemiology, A.O.U. Città della Salute e della Scienza di Torino, Torino, Italy

**Keywords:** biomarkers, CMV, extracellular vesicles, haploidentical hematopoietic stem cell transplantation, viral infections

## Abstract

**Introduction:**

Human cytomegalovirus (CMV), Epstein–Barr virus (EBV), and human herpesvirus 6 (HHV-6) remain important causes of morbidity after allogeneic hematopoietic cell transplantation (HCT), yet biomarkers predicting viral reactivation are lacking. Extracellular vesicles (EVs) have emerged as promising liquid-biopsy tools in inflammatory and infectious diseases.

**Methods:**

In this exploratory biomarker discovery study, 32 consecutive recipients of haploidentical HCT with post-transplant cyclophosphamide were prospectively enrolled. EV surface marker profiles were assessed by flow cytometry before transplantation and at predefined time points during the first three months post-HCT. Associations between EV phenotypes and viral reactivation were evaluated using logistic regression and time-varying Cox models.

**Results:**

CMV, EBV, and HHV-6 reactivation occurred in 20, 11, and 17 patients, respectively. Multivariable analyses showed that different EV phenotypic patterns were associated with CMV, EBV, and HHV-6 reactivation. CMV reactivation was associated with increased EV expression of CD44, CD86, CD140a, and CD144. EBV reactivation correlated with reduced CD30-positive EV concentrations and increased KRT18 expression. HHV-6 reactivation was associated with altered CD26, CD86, CD120a, and CD144 EV profiles. These associations remained significant after adjustment for acute graft-versus-host disease and concomitant viral infections.

**Discussion:**

Collectively, these exploratory findings support further validation of circulating EV phenotypes as candidate biomarkers for predicting and monitoring viral reactivation after haploidentical HCT.

## 1 Introduction

Haploidentical hematopoietic stem cell transplantation (hHCT) with post-transplant cyclophosphamide (PT-Cy) is an effective and widely accessible curative option for patients with high-risk hematologic malignancies. The adoption of reduced-intensity conditionings has expanded its feasibility to older or medically unfit individuals (1). However, viral infections cause significant morbidity, particularly reactivation of latent herpesviruses. Among these, human Cytomegalovirus (CMV) represents the most clinically relevant pathogen, contributing to transplant-related complications, including multi-organ disease, graft dysfunction, and increased susceptibility to secondary infections (2, 3). Several risk factors predispose to CMV reactivation, including immunosuppression intensity, donor–recipient serostatus, conditioning regimen, T-cell depletion strategies, and acute graft-versus-host disease (aGvHD) (4). CMV exhibits broad cellular tropism, infecting endothelial, epithelial, smooth muscle cells, fibroblasts, leukocytes, and dendritic cells (5) through viral complexes, including gH/gL/pUL128-pUL130-pUL131A and gH/gL/gO (5–7).

CMV entry is mediated by several host membrane proteins, including EGFR, integrins, heparan sulfate, THY-1, and particularly PDGFRα (CD140α), which has emerged as a key determinant of viral infection (8, 9). Endothelial cells (ECs) play a central role in CMV pathogenesis, persistence and dissemination, serving as viral reservoirs (10) and exhibiting functional alterations such as increased permeability and upregulation of adhesion molecules including CD44 (11). Other herpesviruses, particularly Epstein-Barr virus (EBV) and Human Herpesvirus 6 (HHV6), also commonly infect or reactivate post-hHCT (12, 13). EBV, a gamma-herpesvirus, displays a dual tropism for B cells and epithelial cells, alternating between latent infection of memory B cells and lytic replication in epithelial tissues. Viral entry involves gp350/220 binding to CD21/CD35, core fusion proteins gH/gL/gB, and gp42-mediated engagement of HLA class II molecules (14–18). HHV6 infects nearly all individuals in early life and persists lifelong with tropism for hematopoietic cells, including CD34+ progenitors (17, 18). It can infect multiple immune cell types and endothelial cells, inducing proinflammatory cytokine release and impairing immune function (19–21). HHV6 reactivation has been associated with delayed engraftment, impaired T-cell reconstitution, increased co-infections, and severe GvHD post-HCT (22–26). Although quantitative PCR monitoring enables early detection of viral replication, biomarkers reflecting the biological processes preceding viral reactivation remain limited (2, 3, 12, 13).

Extracellular vesicles (EVs) have emerged as promising liquid-biopsy tools in inflammation, tissue injury, malignancies, and viral infections (24–31). EVs released by infected cells may carry altered host-derived molecules and potentially reflect early virus-induced perturbations before clinically detectable infection (25–29). We previously generated longitudinal EV immunophenotyping dataset in a cohort of haploidentical HCT recipients to investigate acute GvHD (32). In the present study, we performed a secondary analysis of the same patient cohort, longitudinal sampling schedule, and EV dataset, applying a distinct clinical stratification and analytical framework to investigate the relationship between EV phenotypes and CMV, EBV, and HHV-6 reactivation after transplantation. Specifically, we sought to determine whether circulating EV signatures reflect virus-associated biological pathways and whether they may serve as early indicators of subsequent viral reactivation, thereby laying the groundwork for the development of novel biological risk-stratification strategies in the post-transplant setting.

## 2 Materials and methods

### 2.1 Patients, transplant characteristics, viral infection/reactivation

Thirty-two consecutive patients undergoing hHCT were enrolled at the Bone Marrow Transplant Unit, Humanitas Cancer Center, Humanitas Research Hospital, Rozzano, Milan, Italy, before the introduction of Letermovir in Italy (2019). All patients and donors provided written informed consent. Plasma samples were prospectively collected under institutional biological repository guidelines and stored for EV analyses (**Figure 1**). Patient and transplant characteristics are summarized in **Table 1**. GvHD prophylaxis consisted of PT-Cy 50 mg/kg on days +3 and +4, tacrolimus and/or cyclosporin A, and mycophenolate mofetil (MMF) from day +5. Granulocyte colony-stimulating factor (G-CSF) was started on day +5. Disease response followed the European Bone Marrow Transplantation (EBMT) Group criteria. To identify patients experiencing CMV, EBV, and/or HHV-6 infection/reactivation post-hHCT, viral loads were tested weekly in whole blood (PB) using real-time PCR (CMV R-GENE, EBV R-GENE, HHV-6 R-GENE, Argene, Biomérieux). For study purposes, reactivation was defined as DNA exceeding 1000 IU/mL post-transplantation. This threshold captures early viral replication and may differ from thresholds used in routine clinical practice. CMV pre-emptive antiviral therapy (Foscarnet 90 mg/kg, Ganciclovir 5 mg/kg, or Valganciclovir twice daily for 2 weeks) was initiated above 4000 IU/mL viral load. No pre-emptive therapy was administered for EBV or HHV-6 unless clinically indicated. GvHD was diagnosed according to the Glucksberg grading system.

**Figure 1:**
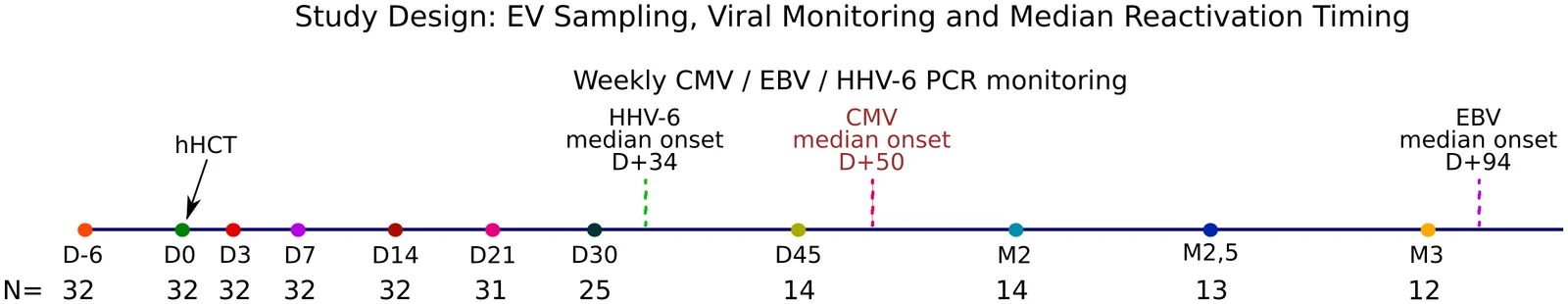
Study design and timing of extracellular vesicle sampling, virologic monitoring, and viral reactivation events. Peripheral blood samples for extracellular vesicle (EV) analyses were collected before transplantation (day −6), at transplantation (day 0), and at predefined time points after haploidentical hematopoietic cell transplantation (hHCT) with post-transplant cyclophosphamide (days +3, +7, +14, +21, +30, +45, and months 2, 2.5, and 3). The number of evaluated patients (N) at each time point is indicated below the timeline. Patients underwent routine weekly quantitative PCR monitoring for cytomegalovirus (CMV), Epstein–Barr virus (EBV), and human herpesvirus 6 (HHV-6) throughout the post-transplant period. Dashed vertical lines indicate the median time to viral reactivation observed in the study cohort: day +34 for HHV-6, day +50 for CMV, and day +94 for EBV. The image illustrates the temporal relationship between EV sampling, virologic surveillance, and the occurrence of viral reactivation events analyzed in this exploratory study. The schedule of EV assessments was aligned with routine weekly virologic surveillance, which also served as the basis for the 7-day interval analyses.

**Table 1:** Patient and transplant characteristics.

N	Sex: d/r	r: age	d: age	Disease	Conditioning regimen	Stem cell source	aGvHD Gluk grade; day post-hHCT	CMV react; day post-hHCT		EBV react; day post- hHCT	HHV6 react; day post-hHCT
11	F	34	29	HL	RIC, Baltimore	BM	2; 33		0	1; 84	1; 40
12	F	44	23	NHL	RIC, Baltimore	BM	0		0	0	1; 39
13	M	54	46	NHL	RIC, Baltimore	BM	1; 92		1; 50	0	0
14	F	57	24	HL	RIC, Baltimore	BM	0		0	0	1; 31
15	F	57	62	NHL	RIC, Baltimore	BM	2; 68		1; 48	0	1; 40
16	F	24	60	HL	RIC, Baltimore	BM	0		0	0	0
17	M	24	45	HL	RIC, Baltimore	BM	0		1; 49	1; 94	0
18	M	25	51	NHL	RIC, Baltimore	BM	1; 40		0	1; 103	0
19	M	47	38	HL	RIC, Baltimore	BM	0		1; 33	1; 54	0
20	M	51	50	NHL	RIC, Baltimore	BM	0		1; 51	1; 216	0
21	F	28	32	HL	RIC, Baltimore	BM	0		1; 275	0	0
22	M	25	21	HL	RIC, Baltimore	BM	1; 75		0	1; 89	0
23	F	26	54	HL	RIC, Baltimore	BM	0		0	0	0
25	M	62	35	NHL	RIC, Baltimore	BM	2; 34		1; 63	0	1; 34
26	F	42	49	NHL	RIC, Baltimore	BM	1; 30		1; 65	1; 314	0
27	F	40	48	HL	RIC, Baltimore	BM	0		1; 29	1; 122	0
28	F	21	52	HL	RIC, Baltimore	BM	1; 41		0	1; 55	0
29	M	24	51	HL	RIC, Baltimore	BM	0		1; 53	0	0
30	F	27	43	HL	RIC, ONC005	BM	1; 57		0	0	0
32	M	32	57	HL	RIC, ONC005	BM	0		1; 29	0	0
33	M	37	47	HL	MAC, TFB	BM	0		0	0	1; 40
35	M	30	22	HL	RIC, Baltimore	BM	0		1; 53	0	1; 30
36	F	66	71	HL	RIC, ONC005	BM	0		1; 56	0	0
38	M	44	27	NHL	MAC, TBF	BM	0		1; 42	0	1; 18
39	M	50	44	AML	MAC, TTFBx3	PBSC	0		0	0	1; 11
40	M	55	56	HL	RIC, ONC005	BM	4; 49		1; 54	0	1; 34
41	F	37	71	NHL	RIC, ONC005	BM	0		1; 34	0	1; 22
42	F	46	35	ALL	MAC, TBF	BM	2; 92		1;102	1; 147	1; 280
44	M	61	36	NHL	RIC, Baltimore	BM	2; 38		1; 10	0	1; 14
46	M	50	46	CLL	RIC, Baltimore	BM	0		1; 153	0	1; 138
47	F	47	45	NHL	RIC, Baltimore	BM	2; 41		1; 50	0	1; 50
48	M	24	51	ALL	MAC, TBF	BM	1; 80		0	1; 55	1; 13

d, donor; r, recipient; HL, Hodgkin Lymphoma; NHL, non-Hodgkin Lymphoma; AML, Acute Myeloid Leukemia; ALL, Acute Lymphoid Leukemia; CLL, Chronic Lymphoid Leukemia; RIC, Reduce Intensity Conditioning; MAC, Myeloablative Conditioning; Baltimore, Fludarabine (30mg/m2; days -6, -5, -4, -3, -2), Cyclophosphamide (14, 5 mg/Kg; days -6, -5), Total Body Irradiation (200 cGy); TBF, Thiotepa (5 mg/Kg; days -6, -5), Fludarabine (50 mg/m2; days -4, -3, -2), Busulphan (30 mg/Kg; days -4, -3, -2); ONC005, Thiotepa (5 mg/Kg twice a day; day -6), Fludarabine (30mg/m2; days -5; -4, -3, -2), Cyclophosphamide (30 mg/Kg; days -5); BM, Bone Marrow; PBSC, Peripheral Blood Stem Cells; aGvHD, acute Graft versus Host Disease; CMV react, Cytomegalovirus reactivation; EBV react, Epstein Barr Virus reactivation; HHV6 react, Human Herpesvirus-6 reactivation; 0, no reactivation; 1, reactivation.

### 2.2 Plasma sample collection

PB samples were collected in Lithium-Heparin tubes from recipients before transplant (day -6) and from recipients at different time-points post-transplant (median, **Figure 1**). Plasma samples were obtained after mononuclear cell separation by density-gradient centrifugation (Lympholyte-H, Cedarlane, Burlington, Canada) and stored at -80 °C.

### 2.3 Extracellular vesicle precipitation and characterization

To remove platelet contamination, plasma samples (~1 ml) were thawed on ice and centrifuged at 2000 × g for 40 minutes at 4 °C. EVs were then precipitated (30), resuspended in 150 μL of Roswell Park Memorial Institute (RPMI) medium supplemented with Penicillin (100 U/L), Streptomycin (100 mg/L), Amphotericin B (0.25 mg/L) (EUROCLONE Spa, Pero, MI, Italy), and 10% Dimethyl sulfoxide (DMSO), and stored at -80 °C. All samples underwent a single freeze-thaw cycle before analysis. EV integrity was confirmed by transmission electron microscopy and nanoparticle tracking analysis (NTA). EVs were allowed to adhere to 200-mesh nickel formvar carbon-coated grids (Electron Microscopy Sciences, Hatfield, Pennsylvania, USA) for 10 minutes. Grids were washed with Phosphate-Buffered Saline (PBS, pH 7.4), fixed with 2.5% glutaraldehyde containing 2% sucrose, negatively stained with NanoVan (Nanoprobes, Yaphank, New York, USA), and examined using a Jeol JEM 1400 Flash electron microscope (Tokyo, Japan). EV size and concentration were assessed by NTA. EV preparations were diluted (1:3000) in sterile saline solution (0.9% NaCl). Brownian motion under laser illumination was recorded (three 30-second videos) using a NanoSight NS300 system (NanoSight Ltd., Minton Park, United Kingdom). Data were analyzed using Nanosight NTA 3.2 software, applying the Stokes-Einstein equation (31). Western blot analysis was performed on EVs precipitated from plasma samples using PEG/protamine to evaluate EV enrichment and the potential co-isolation of non-vesicular components. EV proteins were isolated by lysis for 30 min at 4 °C in RIPA buffer (20 mM Tris-HCl, 150 mM NaCl, 1% deoxycholate, 0.1% SDS, 1% Triton X-100, pH 7.8) supplemented with 1% phenylmethylsulfonyl fluoride and 1% protease and phosphatase inhibitor cocktail (all from Sigma-Aldrich). Protein concentration was quantified using the BCA Protein Assay Kit (Pierce™, Thermo Fisher Scientific, Waltham, MA, USA). Protein samples (30 µg) were separated under reducing conditions on 4–20% gradient Mini-PROTEAN TGX precast gels (Bio-Rad, Hercules, CA, USA) and transferred onto 0.2-µm nitrocellulose membranes using the Trans-Blot Turbo Transfer System (Bio-Rad). Membranes were blocked for 2 h at room temperature in PBS-T containing 0.1% Tween-20 and 5% bovine serum albumin (BSA). They were then incubated for 18 h at 4 °C with primary antibodies, all purchased from Cell Signaling Technology (Danvers, MA, USA). The primary antibodies used included: rabbit anti-GM130 (as a negative control); rabbit anti-TSG101 and anti-CD63 (as EV-specific markers); rabbit anti-ApoB (to evaluate apolipoprotein contamination). After extensive washing, the membranes were incubated for 1 h at room temperature with the appropriate peroxidase-conjugated secondary antibodies (Invitrogen, Thermo Fisher Scientific). Chemiluminescent signals were detected using ECL substrate (Bio-Rad) and acquired with the ChemiDoc Imaging System (Bio-Rad).

### 2.4 EV immunophenotyping

The complete EV immunophenotyping dataset analyzed in the present study originated from the same experimental cohort and flow cytometry acquisitions previously reported (32). No additional flow cytometry measurements were performed, and no new biomarkers were added to the original EV panels. Instead, the existing longitudinal dataset was reanalyzed using a distinct clinical stratification and statistical framework specifically designed to investigate the relationship between EV signatures and CMV, EBV, and HHV-6 reactivation after hHCT. In the original dataset, EVs were characterized by flow cytometry using fluorescein isothiocyanate (FITC)- or phycoerythrin (PE)-conjugated antibodies (**Supplementary Table S1**).

The antibody panel was predefined in our previous studies (30, 32) and was not specifically developed for viral reactivation. It included markers related to immune activation, endothelial biology, and epithelial injury. Because these biological processes may also accompany post-HCT viral reactivation, the same panel was explored in the present secondary analysis without introducing additional markers. CD9 and CD81 were evaluated as EV-associated tetraspanins to identify tetraspanin-positive events within the detectable EV-enriched fraction; their expression was not considered evidence of a specific exosomal origin. Mouse non-immune isotypic FITC or PE IgGs served as negative controls (**Supplementary Table S1**). Incubation with tagged antibodies (0.8-1.5 µL) and EVs (3 × 10^9^ particles), acquisition on a Guava Instrument (GUAVA easyCyteTM 8, Merck Millipore, Billerica, MA, USA), and gating were performed as previously described (32). Instrument calibration for small-particle detection was performed using Megamix beads (300 nm, 500 nm, and 3 µm), Megamix-Plus SSC beads (160 nm, 200 nm, 240 nm, and 500 nm), Nanosight-certified size calibration beads (100 nm, 200 nm, and 400 nm), and Apogee beads (110 nm, 590 nm, and 880 nm) to evaluate sub-micron scatter performance. Based on these calibration experiments, the approximate bead-based lower detection threshold was estimated to be 100–150 nm. Because reference beads and biological EVs differ in refractive index and light-scattering properties, this value does not represent an absolute EV size cutoff. The flow cytometric analysis was therefore expected to detect predominantly larger EVs and a proportion of small EVs, whereas the smallest EVs may have remained below the detection threshold (**Supplementary Figure S1**). For each marker, 5000–10000 events were acquired. Samples were appropriately diluted in filtered FACS flow dilution buffer to ensure a low acquisition speed (<300 events per second) and avoid swarming. Measurements were performed in 2–4 replicates to determine mean fluorescence intensity (MFI), percentage and concentration of marker-positive EVs (**Figure 2**; **Supplementary Figure S2**) (32). Concentration of positive EVs was calculated by multiplying the frequency of marker-positive EV by the total EV concentration measured by NTA. Data were analyzed using GuavaSoft InCyte 2.5 software.

**Figure 2:**
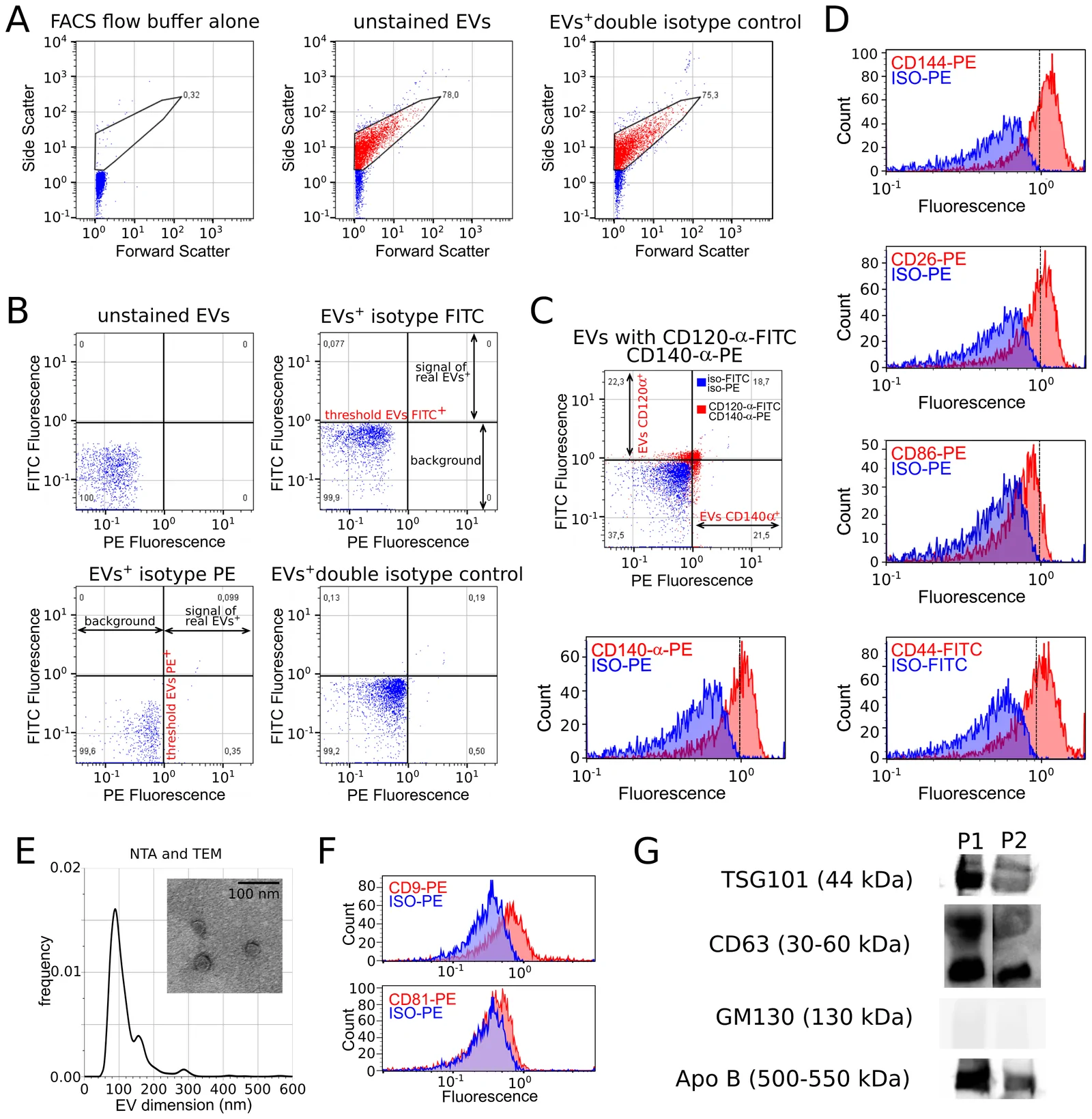
Extracellular vesicle characterization. **(A)** Representative physical parameter dot plots (side scatter versus forward scatter) displaying the gating strategy for extracellular vesicle (EV) isolation: FACS flow buffer alone used to identify background debris (left panel), unstained EVs (middle panel), and EVs incubated with a double isotype control to define the EV-enriched region (gate region on plots). **(B)** Representative fluorescence dot plots (FITC versus PE) used to establish positivity thresholds, showing unstained EVs, EVs incubated with FITC isotype control, EVs incubated with PE isotype control, and EVs incubated with the double isotype control. **(C)** Expression of CD120α and CD140α following incubation with anti-CD120α–FITC and anti-CD140α–PE (red dots) compared with isotypic controls (blue dots), accompanied by the corresponding fluorescence histogram for CD140α expression. Black lines indicate the thresholds used to discriminate CD120α^+^ and CD140α^+^ EVs from background. **(D)** Representative flow cytometry histograms showing fluorescence shifts of EVs stained with the indicated antibodies (red peaks) versus isotypic controls (blue peaks). **(E)** Representative histogram showing EV size distribution assessed by nanoparticle tracking analysis. Inset: representative transmission electron microscopy image of EVs (magnification ×50,000; scale bar, 100 nm). **(F)** Representative histograms showing fluorescence shifts after incubation with tetraspanins markers CD9 (upper panel) and CD81 (lower panel) (red peaks) compared with isotypic controls (blue peaks).CD9 and CD81 positivity identifies tetraspanin-positive EV events within the detectable fraction and does not establish a specific exosomal origin. **(G)** Representative Western blot analysis of EV lysates isolated from 2 patients’ plasma (P1 and P2) via PEG/protamine precipitation, showing the expression of positive EV markers (TSG101 and CD63) alongside an assessment of co-isolated non-EV contaminants, such as apolipoprotein B.

### 2.5 Statistical analyses

For the present secondary analysis, the existing EV dataset was reclassified according to CMV, EBV, and HHV-6 reactivation outcomes and analyzed using the virus-specific statistical framework described below. Cumulative incidences of CMV, EBV, and HHV-6 reactivation were calculated from the transplant date to the onset, considering relapse or death from any cause as competing events, as described by Gooley et al. (33). Patients alive without viral reactivation were censored at the last follow-up. Effects of repeated measurements of each marker on the incidence of viral infection/reactivation were analyzed by dividing patient follow-up into 7-day periods without distinguishing post-transplant time phases. Patients were classified according to presence or absence of viral infection/reactivation during each period. Seven-day intervals were chosen to align EV measurements with the routine weekly virologic surveillance schedule adopted after transplantation (**Figure 1**) and to reduce variability related to non-uniform sampling times. For weeks with multiple measurements of a given marker, the mean value was used. Missing EV measurements were not imputed and analyses were performed using all available observations. The probability of viral infection/reactivation within each 7-day period relative to marker levels (absolute values and changes from pre-transplant), was estimated using logistic regression models (LRMs). Effects on viral infection/reactivation were reported as standardized Odds Ratios (ORs), representing the effect of a 1-standard deviation (SD) increase in the variable, with corresponding p-values. Standard errors were adjusted using the Huber-White sandwich estimator to account for repeated observations. For sensitivity analysis, Cox proportional hazard models (CRMs) were performed using EV parameters as time-varying covariates, with results reported as Hazard Ratios (HRs) for a 1-SD increase. Multivariable LRM and CRM models were constructed for CMV, EBV, or HHV-6 as the outcome variable. Biomarkers with a univariate p ≤ 0.2 were entered into the first step; then, variables were removed using a backward stepwise approach based on p≥0.05. To increase robustness, we implemented a bootstrap stepwise selection procedure based on 500 replications across both our logistic regression and Cox proportional hazards regression models. Using this approach, we calculated the percentage of bootstrap iterations in which each biomarker was retained (Ret %), providing an exploratory measure of variable-selection stability. Effects of other viral reactivations and aGvHD on viral infection/reactivation were adjusted for in multivariable models. Exploratory discriminatory performance of correlated biomarkers was evaluated using univariable LRMs and estimating the Areas Under the Receiver Operating Characteristic Curves (AUROCs). Furthermore, to assess and correct for potential optimism in the estimated discriminatory performance, we used bootstrap resampling with 500 iterations to obtain optimism-corrected AUROC estimates and corresponding 95% confidence intervals. Due to the exploratory and hypothesis-generating nature of this study, traditional statistical adjustments for multiple testing were not applied to avoid excessively inflating Type II errors. Consequently, all statistical comparisons are based on nominal p-values and must be interpreted as exploratory. Mean differences between groups at specific time points were evaluated using two-sided t-tests. Monotonic associations between variables prior to viral reactivation and viremia were assessed using Spearman correlation analysis. As an exploratory analysis, separate univariable logistic regression models were used to evaluate associations between selected EV markers and low-level (1,000–4,000 IU/mL) or higher-level (>4,000 IU/mL) viremia, using <1,000 IU/mL as the reference category. All statistical analyses were conducted using STATA 15 (StataCorp LP) and SPSS Statistics 25 (IBM SPSS Statistics).

## 3 Results

### 3.1 Patient characteristics, opportunistic infections and effect of missing samples

CMV reactivation occurred in 20/32 patients (62.5%), with a median onset at day +50 post-hHCT (range 10-275) (**Table 1**). The cumulative incidence of CMV reactivation at day +180 was 46.90% (95% CI: 32.40-67.80%) (**Supplementary Figure S3**). Only 2/32 patients (6.25%) experienced multiple CMV reactivations. EBV and HHV-6 reactivations were observed in 11/32 (34.4%) and 17/32 (53.1%) patients, with median onset at day +94 (range 54-314) and day +34 (range 11-280) post-hHCT, respectively (**Table 1**). The cumulative incidence at day +180 was 28.13% (95% CI: 16.16-48.9%) for EBV and 43.75% (95% CI: 29.54-64.80%) for HHV-6 (**Supplementary Figure S3**). AGvHD requiring systemic therapy occurred in 7/32 patients (22%), with a median onset at day 41 (range 33-90) (**Table 1**). A total of 8 patients died during the study before completing the three-month sample collection. The missingness rate was calculated up to the median time of reactivation for each virus (or the corresponding median day for patients without reactivation). For CMV (median time 50 days post-hHCT), 26 out of 261 expected samples were missing, yielding a pre-onset missingness rate of 9.96%. Similarly, for HHV-6 (median time 34 days post-hHCT), 25 out of 243 expected samples were missing, resulting in a pre-onset missingness rate of 10.29%. For EBV, which exhibited a longer median time to reactivation (94 days post-hHCT), 79 out of 347 expected samples were missing, leading to a higher pre-onset missingness rate of 22.77%.

### 3.2 EV surface markers associated with CMV reactivation

Univariate analysis did not reveal any association between CD146 and CMV reactivation (**Supplementary Table S2**). CD8 and CD25 were excluded from further analyses due to their low expression on EVs (**Supplementary Figure S2**). Multivariable stepwise selection analysis showed that several EV surface markers were associated with CMV reactivation early post-hHCT, with some biomarkers showing good stability across bootstrap iterations (Ret % ≥ 50) (**Table 2**). LRMs in 7-day intervals showed that CD86 MFI post-hHCT was significantly associated with an increased CMV reactivation risk (OR 1.609, p=0.012). CD140α and CD44 MFIs from day +7, and CD140α and CD144 MFIs from day +14, were also associated with CMV reactivation (OR 1.466, p=0.006; OR 1.373, p=0.012; OR 1.821, p=0.007; OR 1.777, p=0.012, Ret % =31.2, respectively). Changes in CD144 and CD86 MFIs from pre-transplant levels were significantly associated with CMV reactivation in CRMs (HR 2.232, p=0.001, and HR 1.627, p=0.002, respectively) (**Table 2**). Kinetic analysis confirmed that: CD44 and CD144 MFIs were higher at the pre-HCT baseline and remained higher during the evaluated time windows preceding CMV reactivation in patients who subsequently developed CMV reactivation; CD140α MFI was higher in CMV-reactivating recipients 15–30 days before reactivation; proportional changes in CD86 fluorescence were significantly increased 30–45 days before CMV onset (**Figure 3A**; **Supplementary Figure S4**). Spearman’s rank-order correlation showed that CD144 (rho 0.743, p=0.002) and CD86 (rho 0.607, p=0.016) MFIs and the CD86+ (rho 0.593, p=0.020) EV concentration positively correlated with CMV viremia (**Supplementary Table S2**). Patients experiencing CMV reactivation also had higher concentrations of EVs of endothelial origin (CD31+, CD140α+, CD144+, CD146+) (**Supplementary Figure S5**). Multivariable LRMs adjusted for aGvHD and EBV/HHV-6 infections showed that these factors did not affect the association between EV biomarkers and CMV reactivation (**Table 3**).

**Table 2:** Multivariable association analyses between EV surface marker levels and viral infections/reactivations, with bootstrap-based assessment of marker-selection stability across post-HCT time periods.

		Logistic regression model									COX regression model		
		*Post-hHCT*			*From +7d* *post-hHCT*			*From +14d* *post-hHCT*					
*Marker*	*Type*	*OR*	*p*	*%*	*OR*	*p*	*%*	*OR*	*p*	*%*	*HR*	*p*	*%*
CMV													
CD44	MFI	/	/	/	1.373	.012	36.8	/	/	/	/	/	/
CD86	MFI	1.609	.012	50.2	/	/	/	/	/	/	/	/	/
	Δ MFI	/	/	/	/	/	/	/	/	/	1.627	.002	53.8
CD140α	MFI	/	/	/	1.466	.006	65.8	1.821	.007	63.4	/	/	/
CD144	MFI	/	/	/	/	/	/	1.777	.012	31.2	2.232	.001	44.8
EBV													
CD30	Δ Conc	.573	.007	43	.545	.002	86.4	.613	.015	74	.508	.024	74.4
KRT18	MFI	/	/	/	1.595	.028	66.8	/	/	/	/	/	/
	Δ MFI	/	/	/	/	/	/	/	/	/	2.328	.009	83.4
HHV6													
CD26	MFI	1.361	.006	30.0	/	/	/	/	/	/	/	/	/
CD86	MFI	.596	.010	50.6	/	/	/	/	/	/	/	/	/
CD120α	MFI	/	/	/	1.430	.026	24.6	1.513	.048	29.8	1.943	.014	76.2
CD144	Δ Conc	/	/	/	.672	.051	34.6	.629	.045	63.6	/	/	/

Marker analysis was performed using a stepwise backward selection procedure applied to both logistic regression models (evaluating by 7-day period windows: overall post-HCT, or landmark periods starting from +7 or +14 days post-hHCT) and time-varying Cox proportional hazards regression models. The stability of marker selection was explored using a bootstrap-coupled stepwise selection procedure with 500 replications. Odds Ratios (OR), Hazard Ratios (HR), and corresponding p-values are reported for the selected markers, alongside the exact retention percentage (%) across the bootstrap iterations.

hHCT, haploidentical Hematopoietic Stem Cell Transplantation; d, days; OR, odd ratio; HR, hazard ratio; MFI, Mean Fluorescence Intensity; Δ, change with respect to the pre-transplant; Conc, concentration of positive EVs (particles/ml of plasma); %, retention percentage across thebootstrap iterations.

**Figure 3:**
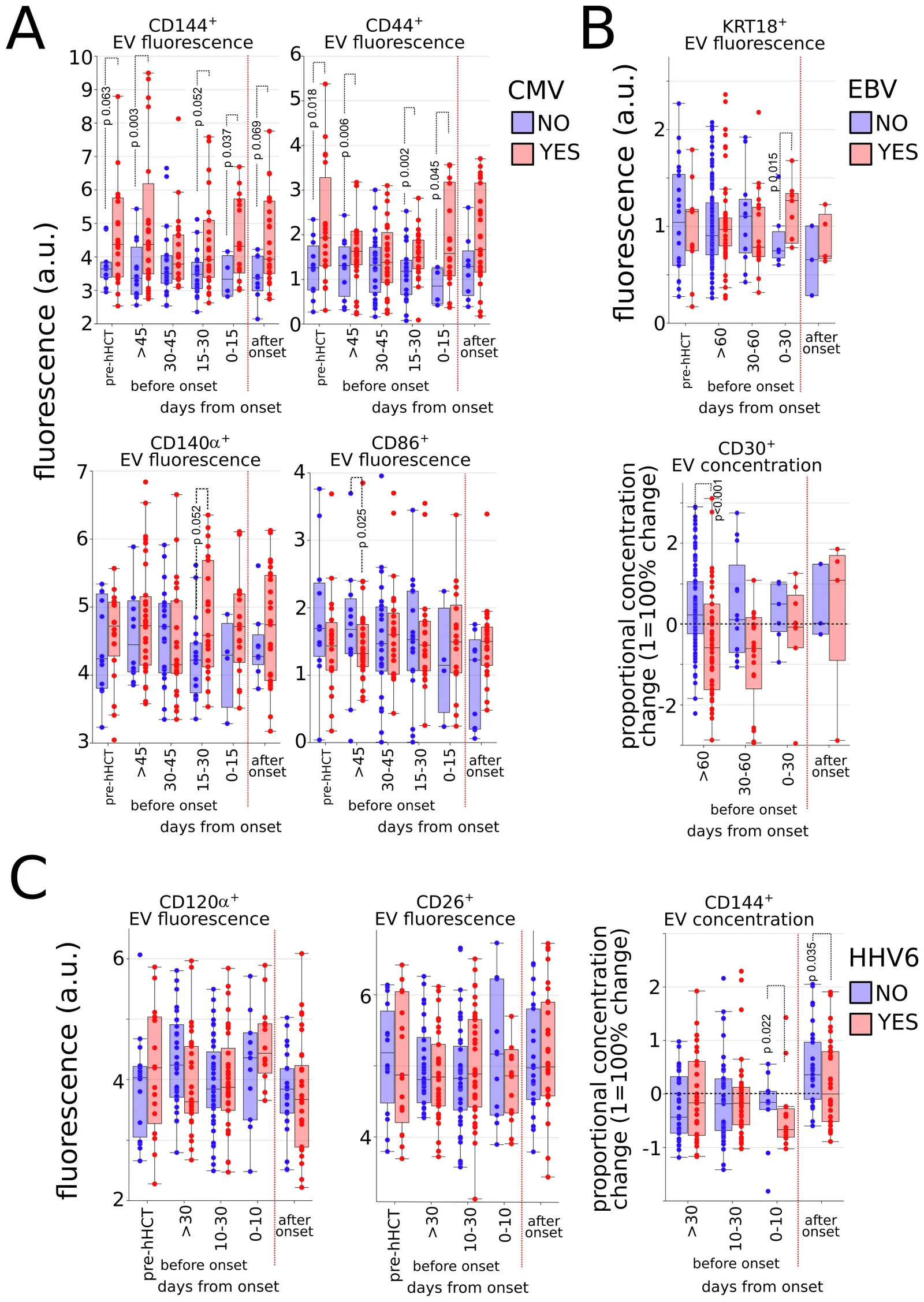
Kinetics of EV membrane protein expression in relation to CMV, EBV, and HHV-6 infection/reactivation. Absolute fluorescence levels and proportional fluorescence changes relative to the pre-transplant baseline (pre-hHCT) are shown for selected biomarkers before and after the onset of **(A)** CMV, **(B)** EBV, and **(C)** HHV-6 infection/reactivation in patients experiencing (red) or not experiencing (blue) viral reactivation. Individual patient data are overlaid as scatter points to show the exact distribution. The dotted red line indicates the time of viral infection/reactivation onset. Significant mean differences between groups (two-sided, p ≤ 0.05) are indicated.

**Table 3:** Effect of opportunistic viral infections/reactivations and aGvHD on biomarker association.

		Multivariable						Univariate	
CMV									
*Marker*	*Type*	CMV-aGvHD		CMV-EBV		CMV-HHV6		CMV	
by LRM		*OR*	*p*	*OR*	*p*	*OR*	*p*	*OR*	*p*
CD44	MFI	1.352	.021	1.328	.025	1.358	.021	1.353	.023
CD86	MFI	1.627	.009	1.644	.009	1.596	.014	1.609	.012
CD140α	MFI	1.941	.011	2.017	.007	1.939	.014	1.881	.007
CD144	MFI	1.848	.009	1.942	.003	1.832	.009	1.777	.012
by CRM		*HR*	*p*	*HR*	*p*	*HR*	*p*	*HR*	*p*
CD86	Δ MFI	1.621	.002	1.643	.002	1.620	.002	1.627	.002
	MFI	2.478	.002	2.484	.001	2.438	.002	1.796	.059
CD144	MFI	2.221	.002	2.325	.007	2.192	.002	2.232	.001

EBV									
*Marker*	*Type*	EBV-aGvHD		EBV-CMV		EBV-HHV6		EBV	
by LRM		*OR*	*p*	*OR*	*p*	*OR*	*p*	*OR*	*p*
CD30	Δ Conc	.544	.001	.545	.002	.499	<.001	.545	.002
KRT18	MFI	1.596	.028	1.597	.028	1.572	.038	1.595	.028
by CRM		*HR*	*p*	*HR*	*p*	*HR*	*p*	*HR*	*p*
CD30	Δ MFI	.444	.013	.493	.020	.530	.037	.508	.024
KRT18	Δ MFI	1.786	.017	1.641	.035	1.596	.036	2.328	.009

HHV6									
*Marker*	*Type*	HHV6-aGvHD		HHV6-CMV		HHV6-EBV		HHV6	
by LRM		*OR*	*p*	*OR*	*p*	*OR*	*p*	*OR*	*p*
CD26	MFI	1.352	.008	1.359	.006	1.364	.005	1.361	.006
CD86	MFI	.562	.005	.600	.010	.601	.010	.596	.010
CD120α	MFI	1.456	.021	1.424	.034	1.427	.031	1.430	.026
CD144	Δ Conc	.660	.071	.634	.055	.631	.048	.629	.045
by CRM		*HR*	*p*	*HR*	*p*	*HR*	*p*	*HR*	*p*
CD120α	MFI	1.942	.014	2.009	.014	1.923	.017	1.943	.014

Marker analysis by 7-day time periods logistic regression (LRM) and by a time-varying approach (CRM). ORs and HRs with p>0.05 and <0.1 are underlined.

CMV, Cytomegalovirus; aGvHD, acute Graft versus Host Disease; EBV, Epstein Barr Virus; HHV6, Human Herpesvirus-6; OR, odd ratio (by logistic regression model, LRM); HR, hazard ratio (by Cox regression model, CRM); MFI, Mean Fluorescence Intensity; Δ, change with respect to the pre-transplant; Conc, concentrationof positive EVs (particles/ml of plasma).

### 3.3 EV surface markers associated with EBV reactivation

Univariate analysis showed that expression of CD26, CD30, CD31, CD86, CD120α, CD140α, CD146, and KRT18 on EVs correlated with EBV reactivation (**Supplementary Table S3**). CD8 and CD25 were excluded due to low expression (**Supplementary Figure S2**). In multivariable stepwise selection models, only CD30 and KRT18 remained associated with EBV reactivation (**Table 2**). CD30 changes on circulating EVs relative to baseline were correlated with EBV reactivation in both LRMs and CRMs, with a proportional concentration decrease of CD30 observed early after hHCT in patients (**Figure 3B**). CD30 showed good biomarker stability, with a Ret % of 86.4% in the CRM, and 74.0% and 74.4% at days +7 and +14 post-hHCT in the LRM, respectively. KRT18 MFI was associated with an increased risk of EBV reactivation starting from 1-week post-transplant (OR 1.595, p=0.028), which was confirmed via CRMs for its MFI (HR 2.328, p=0.009) (**Table 2**). Kinetic analysis showed increased KRT18 expression 0–30 days before EBV onset (**Figure 3B**; **Supplementary Figure S4**).

Multivariate LRMs indicated that aGvHD and CMV/HHV-6 infections did not affect CD30 and KRT18 association with EBV reactivation (**Table 3**). EBV-reactivating patients displayed lower concentrations of endothelial-derived EVs (CD144+, CD146+, CD140α+, CD31+) (**Supplementary Figure S5**). EBV viremia and EV biomarkers were not significantly correlated (**Supplementary Table S3**).

### 3.4 EV surface markers associated with HHV-6 reactivation

Univariate analysis showed that expression of CD26, CD30, CD44, CD86, CD120α, CD138, CD140α, CD144, CD146, and KRT18 on EVs were correlated with HHV-6 reactivation (**Supplementary Table S4**). In multivariable stepwise selection models, only CD26, CD86, CD120α, and CD144 remained significantly associated (**Table 2**). Low CD86 levels post-hHCT increased HHV-6 reactivation risk (OR 0.596, p=0.010), with CD86 MFI significantly lower 0–10 days before onset (**Supplementary Figure S4**). Conversely, higher CD26 levels post-hHCT increased HHV-6 reactivation risk (OR 1.361, p=0.006). High CD120α fluorescence relative to pre-transplant values increased reactivation risk from 7–14 days post-hHCT in LRMs (OR 1.430, p=0.026; OR 1.513, p=0.048) and CRMs (HR 1.943, p=0.0142) (**Table 2**; **Figure 3C**). In contrast, changes in CD144+ EV concentration correlated with HHV-6 reactivation from 7–14 days post-hHCT (OR 0.672, p=0.051; OR 0.629, p=0.045) (**Table 2**), with reduced levels from 10 days before infection (**Figure 3C**). Multivariate models showed that CD144 concentration and HHV-6 reactivation were only slightly influenced by GvHD and CMV (**Table 3**). Spearman’s analysis revealed a negative correlation between HHV-6 viremia, total EV concentration and most EV biomarkers (except CD44, CD86, CD146, CD8, and CD25), while CD44 EV fluorescence positively correlated with HHV-6 viremia (**Supplementary Table S4**).

### 3.5 Discriminatory performance of EV-marker associations

Bootstrap-based marker retention was used as an exploratory measure of variable-selection stability. For CMV, CD140α showed the most consistent retention across the landmark logistic regression models (63.4–65.8%), whereas CD86 showed intermediate stability (50.2–53.8%) and CD44 and CD144 were retained less consistently. For EBV, CD30 and KRT18 generally showed higher retention across the selected models (66.8–86.4%), although CD30 retention was lower in the overall post-HCT model. For HHV-6, the highest retention was observed for CD120α in the time-varying Cox model (76.2%) and for CD144 in the analysis starting from day +14 (63.6%).

To explore the potential discriminatory ability of the most relevant EV markers, exploratory optimism-corrected AUROC analyses were performed. Given the limited sample size and the absence of an independent validation cohort, these analyses should be considered hypothesis-generating. Among the individual markers evaluated at specific time points, the highest optimism-corrected AUROC estimates were observed for CD44 at day +7 for CMV reactivation, the change in CD30-positive EV concentration at day +14 for EBV reactivation, and CD120α at day +7 for HHV-6 reactivation. Regarding marker combinations, the CD44/CD140α, CD30/KRT18, and CD120α/CD86 pairings demonstrated the highest discriminatory performance for CMV, EBV, and HHV-6 reactivation, respectively (**Table 4**; **Figure 4**).

**Table 4:** Biomarker performance at different times after hHCT.

*Marker*	*Type*	*Post-hHCT*		*From +7d* *post-hHCT*		*From +14d* *post-hHCT*		*Pre-hHCT*		*+7d* *post-hHCT*		*+14d* *post-hHCT*	
CMV													
		AUROC	95%ci	AUROC	95%ci	AUROC	95%ci	AUROC	95%ci	AUROC	95%ci	AUROC	95%ci
CD44	MFI	.52	.501-.564	.575	.511-.639	.574	.506-.651	.521	.502-.684	.834	.714-.984	.58	.504-.689
CD86	MFI	.617	.543-.697	.648	.503-.772	.68	.577-.793	.514	.503-.648	.546	.501-.712	.53	.503-.729
	Δ MFI	.682	.523-.842	.696	.502-.877	.71	.537-.892	.744	.578-.921	.646	.501-.786	.623	.501-.789
CD140α	MFI	.534	.502-.573	.594	.523-.654	.652	.548-.751	.646	.507-.82	.684	.514-.859	.65	.503-.737
CD144	MFI	.56	.506-.62	.629	.545-.723	.65	.554-.742	.537	.503-.639	.666	.515-.832	.515	.501-.722
EBVV													
		AUROC	95%ci	AUROC	95%ci	AUROC	95%ci	AUROC	95%ci	AUROC	95%ci	AUROC	95%ci
CD30	Δ Conc	.685	.552-.838	.696	.568-.839	.676	.547-.816	.71	.518-.89	.647	.507-.85	.784	.639-.988
KRT18	MFI	.532	.502-.576	.586	.508-.672	.582	.505-.673	.58	.505-.692	.688	.528-.885	.554	.505-.667
	Δ MFI	.529	.501-.613	.575	.504-.676	.576	.505-.684	.539	.502-.7	.558	.53-.672	.513	.505-.676
HHV6													
		AUROC	95%ci	AUROC	95%ci	AUROC	95%ci	AUROC	95%ci	AUROC	95%ci	AUROC	95%ci
CD26	MFI	.544	.504-.583	.541	.503-.584	.523	.501-.565	.578	.507-.693	.513	.503-.684	.573	.505-.682
CD86	MFI	.587	.505-.67	.55	.504-.627	.538	.503-.613	.672	.517-.839	.74	.527-.896	.523	.504-.676
CD120α	MFI	.54	.5-.554	.576	.505-.643	.585	.509-.663	.735	.577-.921	.759	.617-.939	.522	.503-.699
CD144	Δ Conc	.652	.518-.795	.666	.526-.81	.687	.538-.844	.537	.503-.639	.599	.506-.725	.65	.505-.74

Individual AUROC (Area Under the Receiver Operating Characteristics) curve analysis by univariate Logistic Regression Model at all time points after hHCT (post-hHCT), starting from 7 or 14 days after hHCT (From +7d/+14d post-hHCT) or in the pre-transplant period (pre-hHCT), at 7 or 14 days post-hHCT (+7d/+14d post-hHCT). To assess and correct for potential optimism, AUROC estimates and corresponding 95% confidence intervals were adjusted using bootstrap resampling. Biomarkers with an optimism-corrected AUROC greater than 0.750 are highlighted in gray.

hHCT, haploidentical Hematopoietic Stem Cell Transplantation; d, days; AUROC, area under the receiver operating characteristic curve; ci, confidence interval; MFI, Mean Fluorescence Intensity; Δ, change with respect to the pre-transplant; Conc, concentration of positive EVs (particles/ml of plasma).

**Figure 4:**
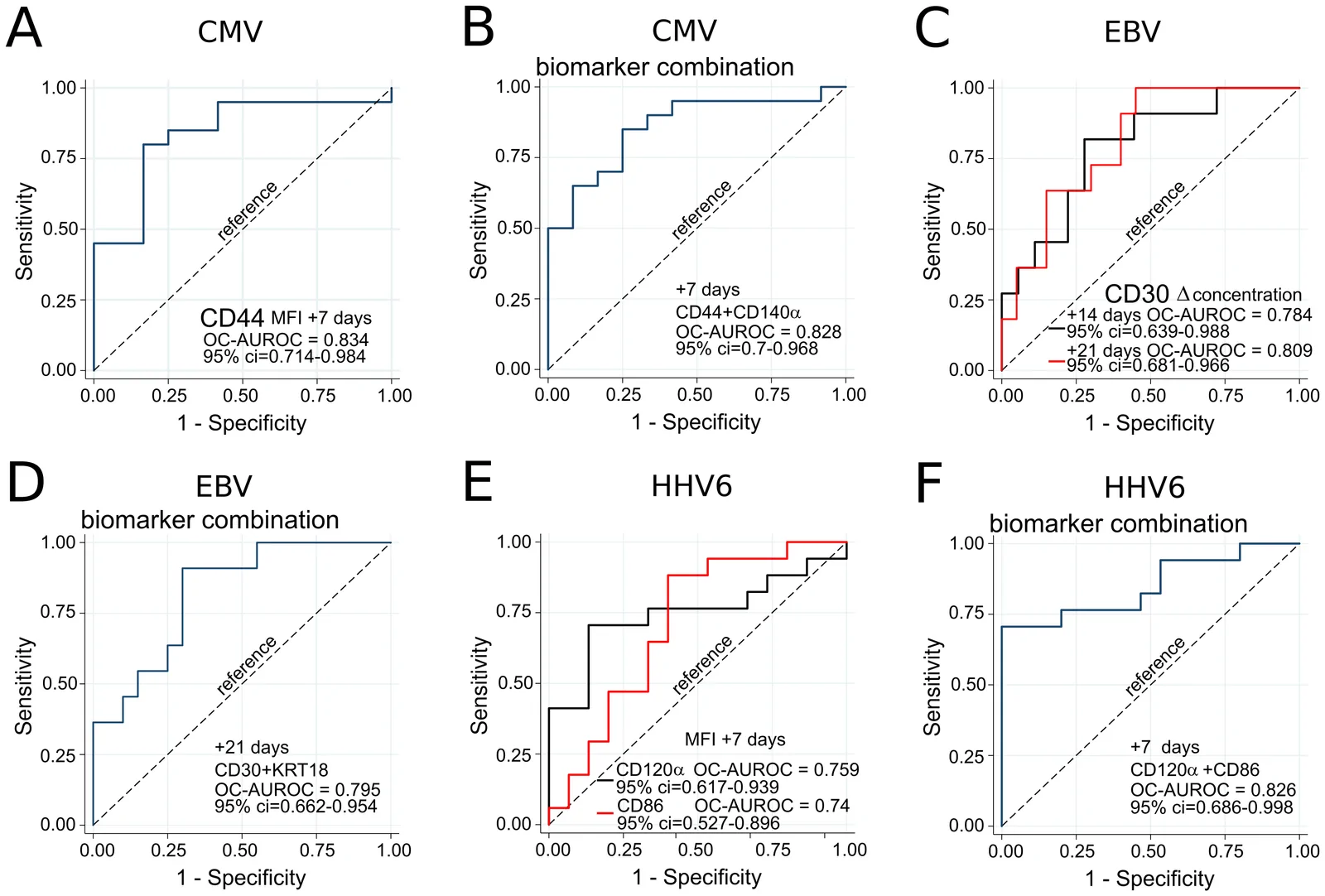
Biomarker discriminatory ability. Upper panels: Exploratory optimism-corrected AUROC analyses with 95% ci for individual biomarkers associated with subsequent viral reactivation. **(A)** Association of CD44 absolute fluorescence (MFI) at day +7 post-hHCT with subsequent CMV reactivation. **(C)** Association of changes (Δ) in CD30^+^ EV concentration at days +14 and +21 post-hHCT relative to pre-transplant baseline with subsequent EBV reactivation. **(E)** Association of CD120α and CD86 absolute fluorescence (MFI) at day +7 post-hHCT with subsequent HHV-6 reactivation. Lower panels: Exploratory multivariable optimism-corrected AUROC analyses with 95% ci showing combinations of EV biomarkers with the highest discriminatory performance. **(B)** CD44 and CD140α absolute fluorescence (MFI) at day +7 for CMV reactivation. **(D)** Changes in CD30^+^ EV concentration relative to pre-transplant baseline combined with KRT18 absolute fluorescence at day +21 for EBV reactivation. **(F)** CD120α and CD86 absolute fluorescence at day +7 for HHV-6 reactivation. OC AUROC, optimism-corrected area under the receiver operating characteristic curve; dotted line, reference line; ci, confidence interval.

### 3.6 Associations according to viral-load level

To assess whether the EV-marker associations identified using the study-defined viral reactivation threshold of >1,000 UI/mL remained detectable at higher levels of viral replication, we performed an exploratory *post hoc* analysis stratifying viral DNAemia into three categories: <1,000 IU/mL (no reactivation), 1,000–4,000 IU/mL (subclinical reactivation), and >4,000 IU/mL (**Supplementary Table S5**; **Supplementary Figure S6**).

For CMV, several of the associations identified in the primary analysis were preserved in patients who subsequently developed viral loads >4,000 IU/mL. Across the entire post-hHCT follow-up, increased CD86 MFI was associated with CMV DNA >4,000 IU/mL (OR 1.630, p=0.019). In analyses starting from day +7, higher CD86, CD140α, and CD144 MFI were associated with CMV DNA >4,000 IU/mL (OR 2.088, p=0.021; OR 1.486, p=0.008; and OR 1.612, p=0.029, respectively), whereas CD44 showed a similar trend that narrowly missed statistical significance (OR 1.309, p=0.058). These association remained evident also at day +14, with CD86, CD140α, and CD144 consistently associated with CMV DNA >4,000 IU/mL (OR 2.276, p=0.014; OR 1.950, p=0.004; and OR 1.901, p=0.005, respectively). Notably, CD86 and CD140α were also associated with CMV DNA levels of 1,000–4,000 IU/mL in the day +14 landmark analysis.

For HHV-6, the overall pattern of associations was broadly consistent with that observed in primary analysis. Lower CD86 MFI was associated with HHV-6 DNA >4,000 IU/mL when considering the entire post-transplant period (OR 0.597, p=0.007). in contrast, higher CD120α MFI and lower CD144-positive EV concentrations were more strongly associated with low-level reactivation (1,000–4,000 IU/mL) both in the day +7 (OR 1.592, p=0.011; and OR 0.427, p=0.006, respectively) and day +14 (OR 1.648, p=0.006; and OR 0.368, p=0.001, respectively) analyses.

For EBV, neither CD30-positive EV concentration nor KRT18 MFI showed statistically significant associations after stratification according to viral-load categories. Because of the limited number of observations within these subgroups, particularly for EBV, the stratified analyses should be considered exploratory.

## 4 Discussion

In this exploratory study, we identified distinct EV immunophenotypic signatures associated with CMV, EBV, and HHV-6 reactivation after haploidentical HCT. Notably, several EV-associated markers showed dynamic changes before clinically detectable viral reactivation, suggesting that EV profiling may capture biological processes involved in host-virus interactions following transplantation. These observations align with previous studies showing that herpesviruses influence EV biogenesis and cargo composition and may exploit EV-mediated pathways to modulate immune responses and facilitate viral persistence or dissemination (25–29, 34–37).

Among the three viruses investigated, CMV displayed the most robust and biological coherent EV signature. Increased expression of CD140α, CD144, CD44, and CD86 was consistently associated with subsequent CMV reactivation across multiple analytical approaches. This profile is biologically plausible given the well-established endothelial tropism of CMV and the recognized role of endothelial cells as viral reservoirs (10). Accordingly, CMV infection and the accompanying inflammatory milieu have been reported to induce endothelial activation and to increase the release of endothelial-derived EV (24, 25, 38, 39). CD140α has been implicated in CMV entry into epithelial and endothelial cells (8, 9), whereas CD144 (VE-cadherin) and CD44 are involved in endothelial junction integrity and vascular homeostasis (40, 41). Collectively, these observations suggest that endothelial activation, cellular stress, or vascular injury may represent early biological events preceding clinically detectable CMV DNAemia.

Intriguingly, elevated CD44 and CD144 were already detectable before the transplantation and persisted throughout the post-transplant period in patients who later developed CMV reactivation. This finding raises the possibility that these markers may reflect a pre-existing host susceptibility state rather than a direct consequence of viral replication alone. Such an interpretation is consistent with the multifactorial nature of CMV reactivation after HCT, where transplant characteristics, immune reconstitution dynamics, endothelial vulnerability, and viral factors jointly contribute to disease risk.

Although causality cannot be inferred from the present data, the CMV-associated EV signature observed herein is supported by a substantial body of experimental evidence linking CMV infection to endothelial dysfunction, altered VE-cadherin organization, and CD44-dependent inflammatory responses (40–44). These findings raise the possibility that the increased abundance of endothelial-associated EV markers may reflect early endothelial perturbation occurring before clinically detectable CMV DNAemia. Potential mechanisms include endothelial barrier remodeling, enhanced leukocyte trafficking, vascular stress responses, and EV-mediated host-pathogen communication. However, the present study was not designed to address these mechanisms directly, and functional studies will be required to determine whether these EV populations merely reflect, or actively contribute to, CMV-associated pathophysiology. Importantly, comparable endothelial EV alterations were not detected during HHV-6 or EBV reactivation, suggesting that the observed signature may be relatively specific to CMV and consistent with the distinct biological tropisms and immune-interaction profiles of these herpesviruses (14, 45).

The stratified viral-load analysis further strengthened the biological relevance of these observations. Several CMV-associated markers, particularly CD86, CD140α, and CD144, maintained the same direction of association among patients who subsequently exceeded 4,000 IU/mL of CMV DNA. Although exploratory, these findings suggest that the identified EV alterations are not restricted to episodes of low-level DNAemia but may remain detectable during higher levels of viral replication that are more likely to influence clinical management.

Beyond endothelial-related markers, our analyses also revealed alterations in EV-associated molecules involved in immune regulation. In particular, both CD26 and CD86 were associated with viral reactivation, although with different levels of statistical support and distinct virus-specific patterns. CD26 (dipeptidyl peptidase-4) is a multifunctional co-stimulatory molecule expressed by activated lymphocytes, endothelial cells, and epithelial cells, and has previously been implicated in antiviral immunity, CMV-specific immune responses, and post-transplant outcomes (46–49).

Although reduced CD26 expression has been reported on CMV- or EBV-specific CD8+ T cells (50), we observed increased EV-associated CD26 following CMV and EBV reactivation. Because this association was not retained in multivariable models, its biological significance should be interpreted cautiously. Increased CD26-positive EVs may reflect broader inflammatory processes, tissue injury, or immune activation accompanying viral reactivation rather than a virus-specific mechanism (51, 52).

In contrast, CD86 emerges as a more robust candidate marker. CD86 plays a central role in antigen presentation and T-cell co-stimulation and is known to be modulated by both CMV and HHV-6 infection (21, 47, 53–57). Interestingly, EV-associated CD86 levels increased in presence of CMV reactivation but decreased in association with HHV-6 reactivation. These opposite patterns may reflect differences in the inflammatory programs and immune-evasion strategies triggered by the two viruses. Although the cellular origin as well as the functional and biological significance of CD86-positive EVs were not investigated, the observed divergence supports the notion that distinct herpesviruses could generate unique EV-associated immune signature that can be detected in the circulation.

Moreover, in contrast to CMV, HHV-6 reactivation was not associated with endothelial EV markers, but was characterized primarily by alterations involving not only CD86 but also CD120α. The selective association of CD120α with HHV-6 is particularly intriguing given the known interactions between HHV-6 and TNF-related signaling pathways (58). Because TNF-α signaling contributes to antiviral immune responses and has been implicated in HHV-6 immune-evasion mechanisms, the identification of EV-associated CD120α may reflect a biologically relevant aspect of host-virus interaction. These observations support further investigation of TNF-related EV signatures in HHV-6 biology following transplantation.

For EBV, the most notable associations involved CD30 and KRT18. CD30 is a member of the TNF receptor superfamily closely linked to lymphocyte activation and EBV-associated lymphoproliferative disorders (59, 60). In our cohort, patients who subsequently developed EBV reactivation exhibited lower concentrations of CD30-positive EVs early after transplantation. One possible explanation is that EBV-driven modulation of ADAM-family metalloproteases alters the balance between EV-associated and soluble CD30 release (61–63). However, because soluble CD30 and metalloproteinase activity were not directly measured, this mechanistic interpretation remains strictly hypothetical. Similarly, increased EV-associated KRT18 was associated with EBV reactivation. KRT18 is a cytokeratin commonly released during epithelial-cell injury and apoptosis and previously linked to gastrointestinal inflammation and GvHD (62–65). Given the epithelial tropism of EBV (66), these findings may be compatible with epithelial cell perturbation occurring during viral reactivation. However, KRT18 lacks virus specificity, and neither the tissue source nor the biological function of KRT18-positive EVs were determined. Consequently, this association should be interpreted primarily as a biomarker signal rather than direct evidence of EBV-induced epithelial damage.

From a translational perspective, the repeated EV assessments performed in this study were primarily intended to characterize biomarker kinetics and identify informative post-transplant time windows, rather than to support intensive EV monitoring in routine clinical practice. We fully acknowledge that quantitative PCR remains the gold standard for the detection and monitoring of viral DNAemia and for guiding clinical decision-making. Rather than replacing molecular diagnostics, EV profiling may provide complementary biological information by identifying patients at increased risk of subsequent clinically significant viral reactivation. If validated in independent prospective cohorts, a limited number of strategically selected post-transplant assessments may be sufficient to capture informative EV signatures and support biological risk stratification alongside standard virological monitoring.

Several limitations of this study must be acknowledged. The investigation was conducted on a small, single-center cohort, limiting statistical power and increasing the risk of overfitting. Furthermore, evaluating multiple EV markers across different time points and analytical models may increase the type I error rate, despite the consistency observed across analyses. The observational design precludes causal inference, and no experiments were performed to determine the cellular origin, molecular cargo, or functional effects of the identified EV populations. Consequently, the mechanistic interpretations proposed here should be regarded as biologically plausible hypotheses requiring validation in dedicated experimental systems. Additionally, while missingness rates for CMV and HHV-6 remained low to moderate (≤10.29%), minimizing the risk of significant clinical bias, the missingness rate for EBV was higher due to the extended follow-up window required to capture its onset; nonetheless, it remains within acceptable limits for long-term longitudinal clinical studies.

To address potential instability issues arising from stepwise selection and absence of external validation, a bootstrap-based retention frequencies and optimism-corrected AUROC analyses were employed. Therefore, while these analyses provide a helpful framework for prioritizing candidate EV biomarkers and informative sampling windows, the clinical utility of the identified EV signatures will require validation in larger, independent prospective cohorts.

An additional methodological consideration concerns the precipitation-based EV isolation procedure. Although this approach is well suited to the limited plasma volumes available in longitudinal transplant studies, it may co-isolate non-vesicular components, including lipoproteins and protein aggregates. To assess EV enrichment, preparations were characterized using complementary techniques, including TEM, NTA, flow-cytometric detection of tetraspanins, and Western blot analysis of both EV-associated and non-EV markers. Nevertheless, residual co-isolated material cannot be completely excluded. Because NTA measures all light-scattering particles and does not discriminate EVs from co-isolated non-vesicular structures, the calculated concentrations of marker-positive EVs may be affected by the presence of non-vesicular particles. Consequently, the analyzed preparations should be considered EV-enriched fractions rather than highly purified EV isolates. Finally, although all samples underwent a single, standardized freeze-thaw cycle, a potential effect on EV integrity and recovery cannot be entirely excluded.

An important consideration for the interpretation of our CMV-related findings is that the study cohort was enrolled before the widespread implementation of Letermovir prophylaxis. Letermovir has substantially altered the incidence, timing, and kinetics of CMV reactivation after HCT and may consequently modify CMV-associated EV signatures. Therefore, whether the biomarkers identified in this study remain detectable or retain similar predictive value in patients receiving active prophylaxis remains unknown. Nevertheless, the present cohort provides a valuable biological reference framework describing EV dynamics during unmodified CMV reactivation. Future studies in contemporary Letermovir-treated populations will be essential to assess the generalizability of these findings.

In conclusion, our findings demonstrate that CMV, EBV, and HHV-6 reactivation are associated with distinct circulating EV signatures after hHCT. The fact that several of these alterations precede measurable viral DNAemia suggests that EV profiling may capture early biological events occurring during host-virus interaction. Although these observations require independent validation and mechanistic investigation, they support further exploration of EV-based biomarkers as complementary tools for biological risk stratification and personalized viral surveillance after transplantation.

## Data Availability

The raw data supporting the conclusions of this article will be made available by the authors, without undue reservation.
