# Supplementary material for: Extracellular vesicle phenotypic patterns associated with CMV, EBV and HHV-6 reactivation after haploidentical transplantation with post-transplant cyclophosphamide
Source: Front Immunol. 2026 Oct 5;17:1937675. doi: 10.3389/fimmu.2026.1937675 (PMC13677358; doi:10.3389/fimmu.2026.1937675)
Supplement: Supplementary file 1 [file DataSheet1.pdf]

Supplementary Material

1 Supplementary Figures and Tables

1.1 Supplementary Figures

Figure S1. Instrument calibration for small-particle detection.
Representative dot plots showing the detection limit of the GUAVA flow cytometer for small particles, using a mixture of beads of known sizes including Megamix beads (300-500nm and 3microm), Megamix-Plus SSC (160-200-240-500nm), polystyrene calibration beads for NanoSight (100-200-400nm), and Apogee beads (110-590-880nm).

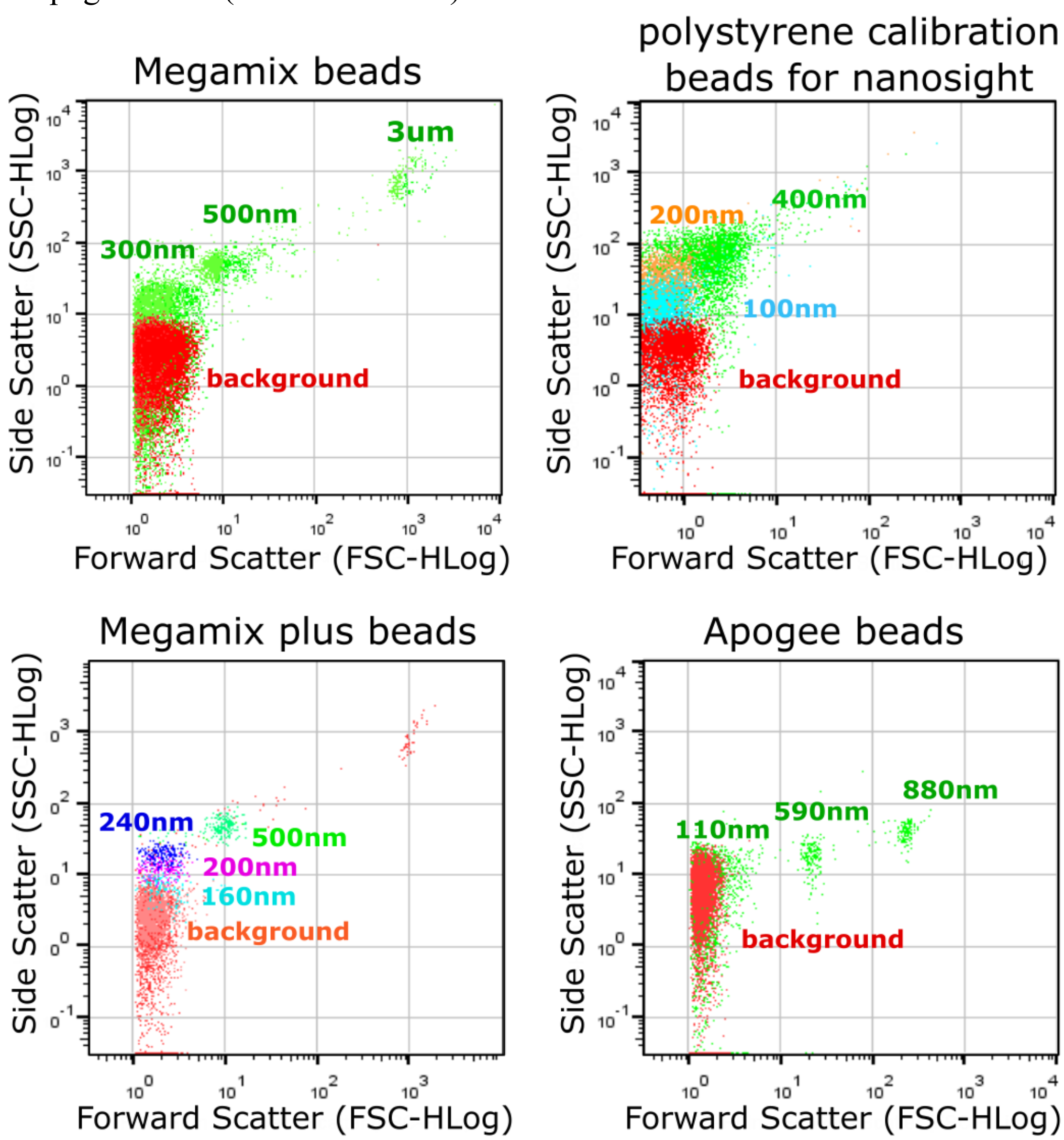

Figure S2. EV fluorescence histograms.
Representative histograms showing the fluorescence shift after incubation of EVs with the indicated antibody conjugated with FITC or PE (blue) with respect to their isotypic control (ISO; red).

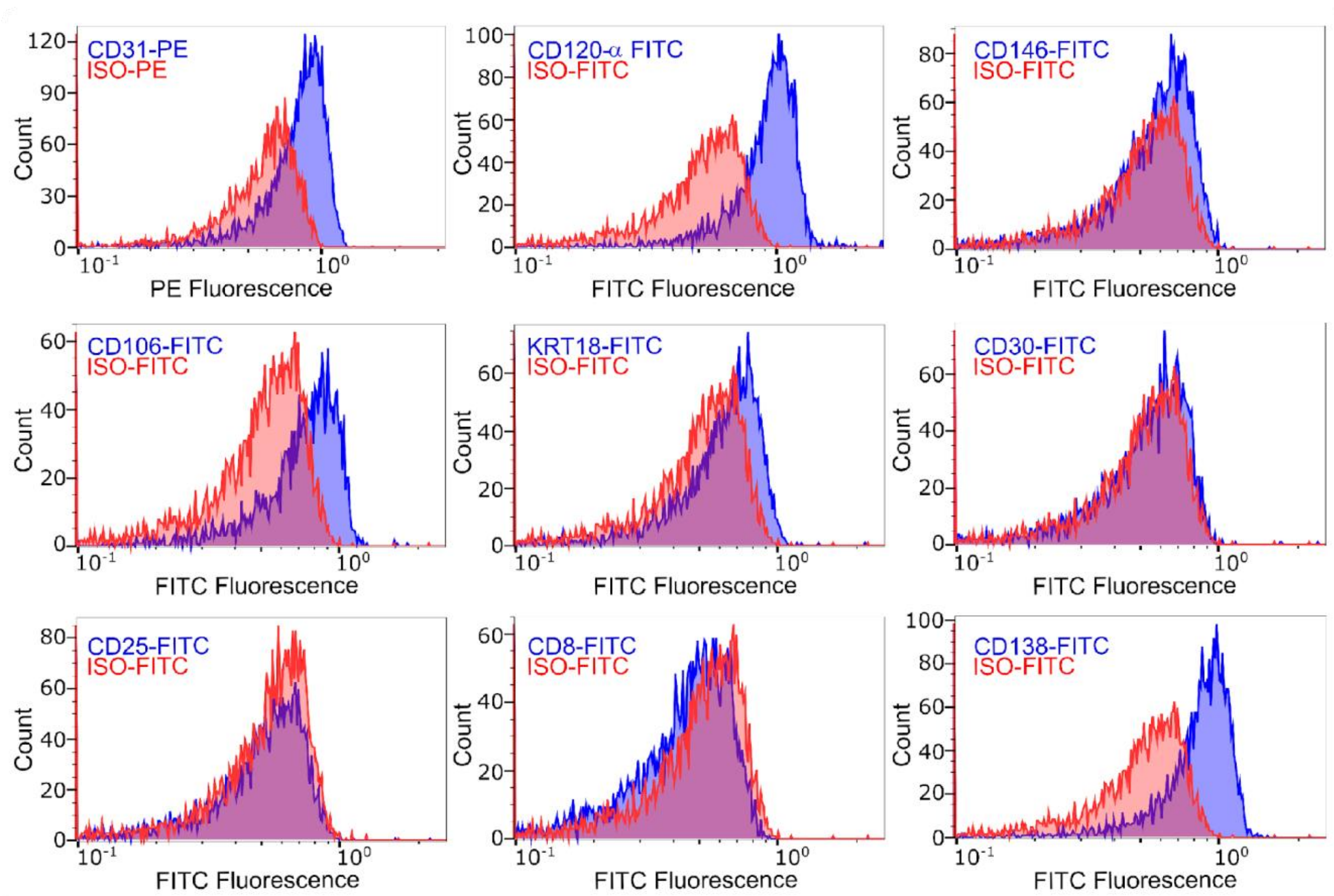

Figure S3. Cumulative incidence of viral infections/reactivations after hHCT.
Cumulative incidence of CMV (left panel), EBV (middle panel), and HHV-6 (right panel) reactivation post-hHCT. The reported cumulative incidence is at 6 months post-hHCT.

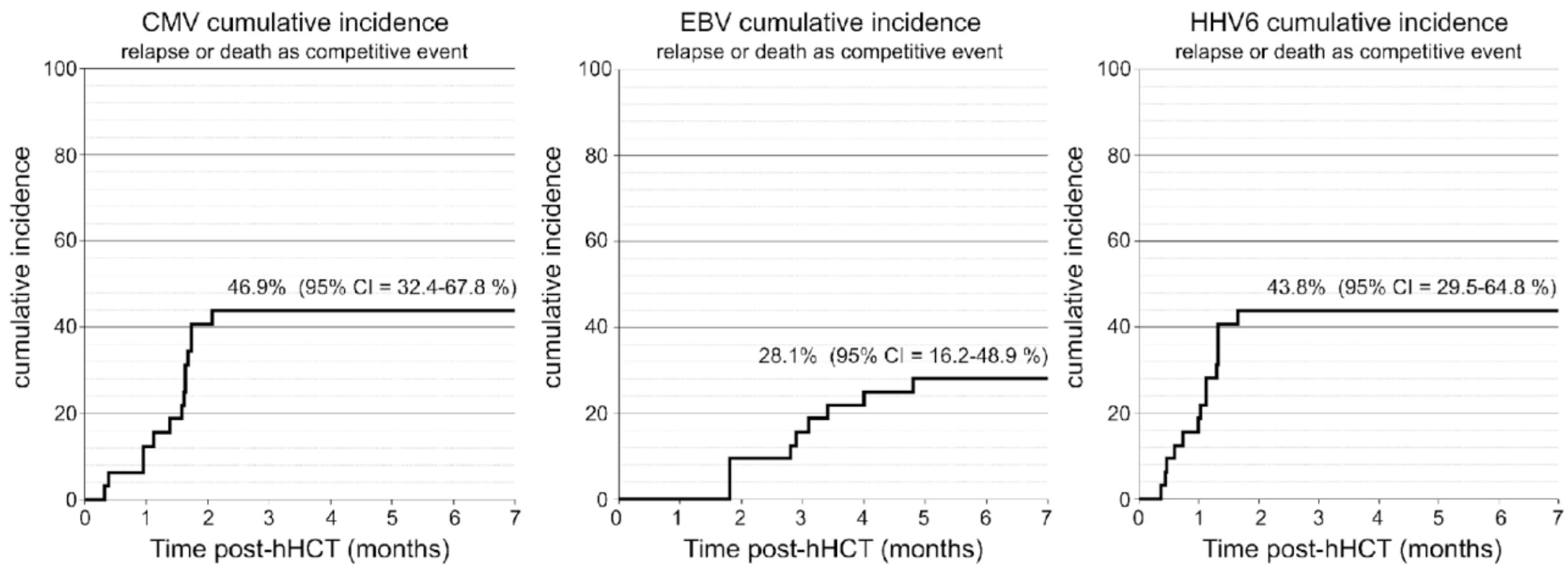

Figure S4. Kinetic of EV membrane protein expression based on the onset of viral infection/reactivation.

Proportional fluorescence changes with respect to the pre-transplant baseline and absolute fluorescence level of the given biomarkers at different time points before and after CMV, EBV and HHV6 infection/reactivation onset in patients experiencing (red) or not (blue) viral reactivations. Individual patient data are overlaid as scatter points to show the exact distribution. Dotted red line represents the time of the onset of viral infection/reactivation event(s). Significant mean differences (2 sides, $p \leq 0.05$) between patients with (red) and without (blue) viral reactivation are indicated.

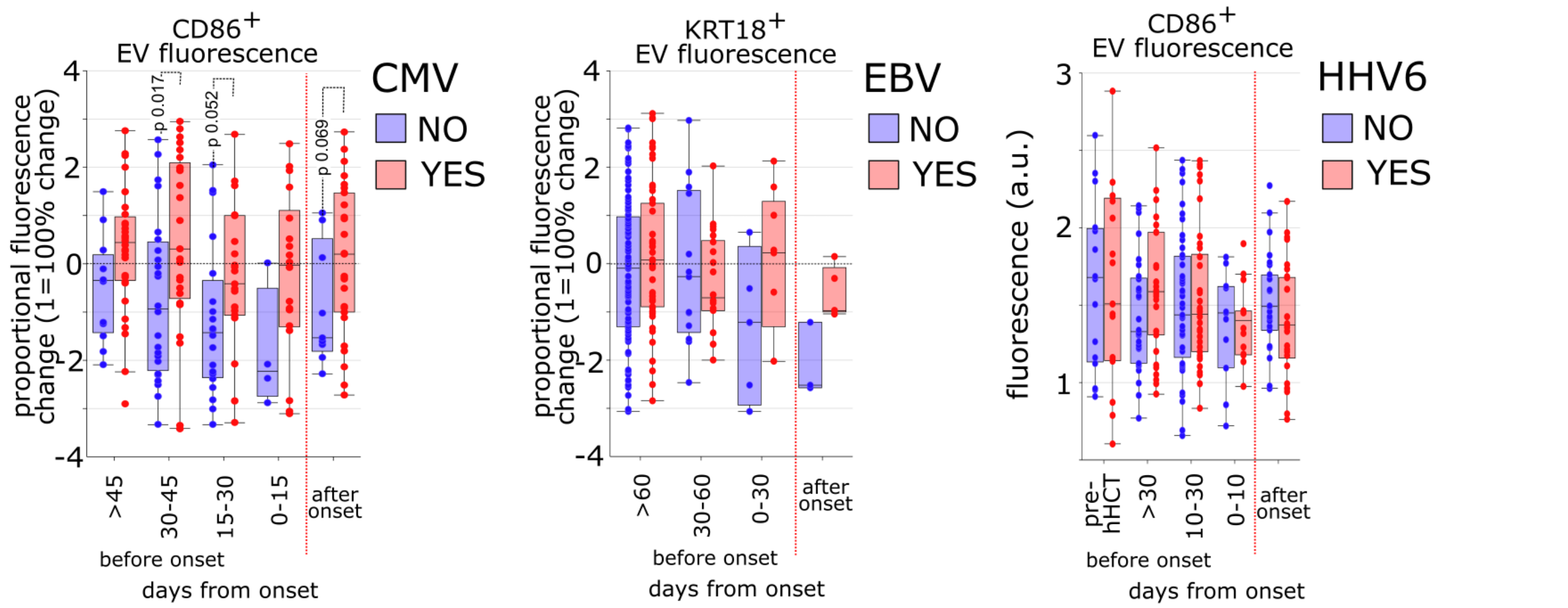

Figure S5. Kinetics of viral and EV levels.

(A, B, C) Kinetic of CMV, EBV and HHV6 viremia (counts/ml) at different time before and after infection/reactivation onset. Green lines represent the viral count sensitivity threshold for low-level viremia (1000) and clinically significant viremia (4000). Dashed red lines represent the onset of infection/reactivation event(s). **(D, E, F)** Standardized concentration level of Endothelial EV particles (1=median standardized concentration level of CD144+, CD146+, CD140α+, and CD31+ EVs) at different time before and after CMV, EBV and HHV6 infection/reactivation onset. Individual patient data are overlaid as scatter points to show the exact distribution. Dashed red lines represent the onset of infection/reactivation event(s). Dashed black lines represent the median concentration level.

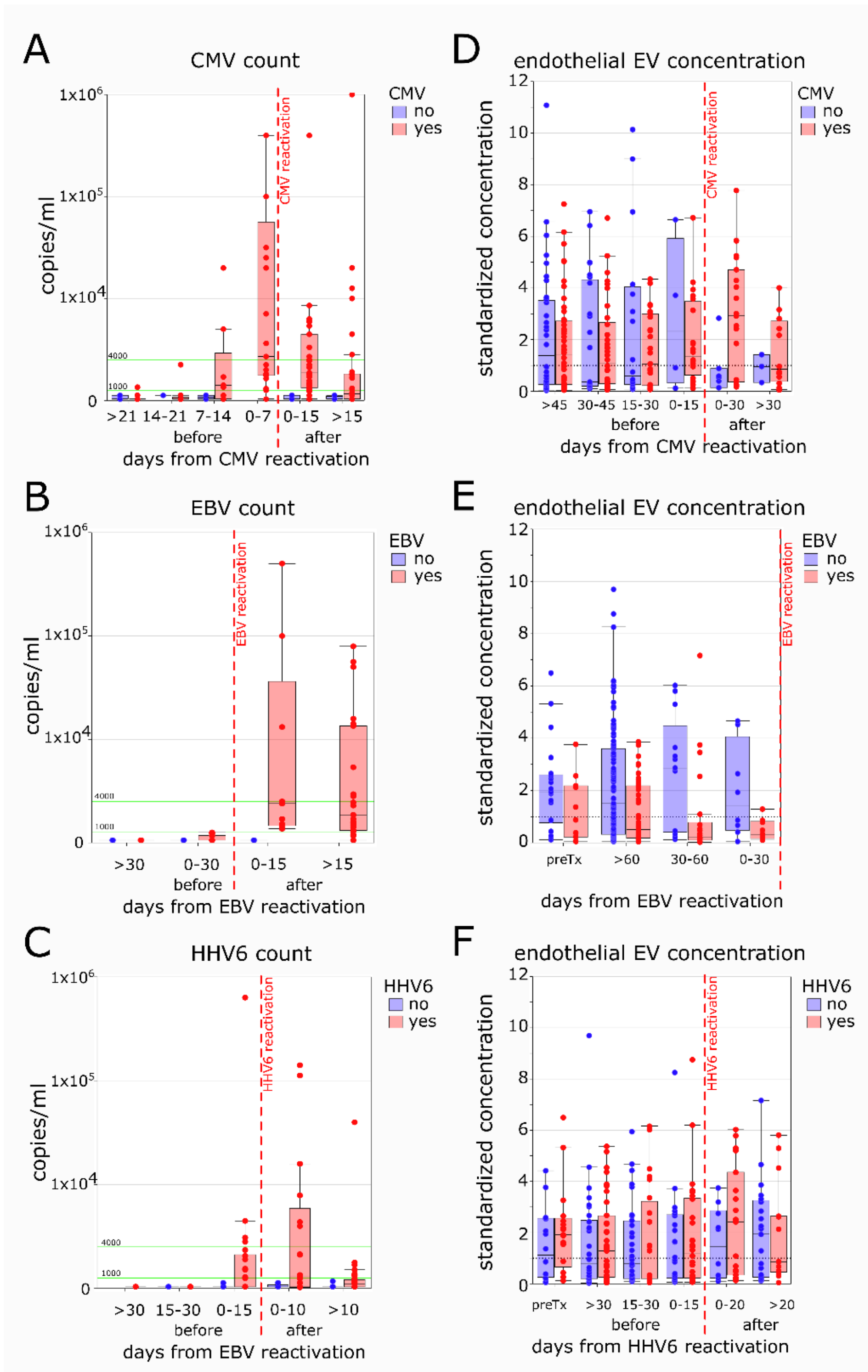

Figure S6. Viral DNA load and EV membrane protein expression.
Absolute fluorescence levels and proportional concentration changes relative to the pre-transplant baseline (pre-hHCT) are shown for selected biomarkers 0–15 days before and after the onset of (A) CMV infection/reactivation and 0–10 days before and after the onset of (B) HHV-6 infection/reactivation. Patients are stratified into three groups: no viral reactivation (blue, viral load < 1000 IU/ml), low-level viremia (orange, viral load 1000–4000 IU/ml), and clinically significant viremia (red, viral load > 4000 IU/ml). Individual patient data within this time range are overlaid as scatter points on the boxplots to show the exact distribution. The dotted red line indicates the time of viral infection/reactivation onset.

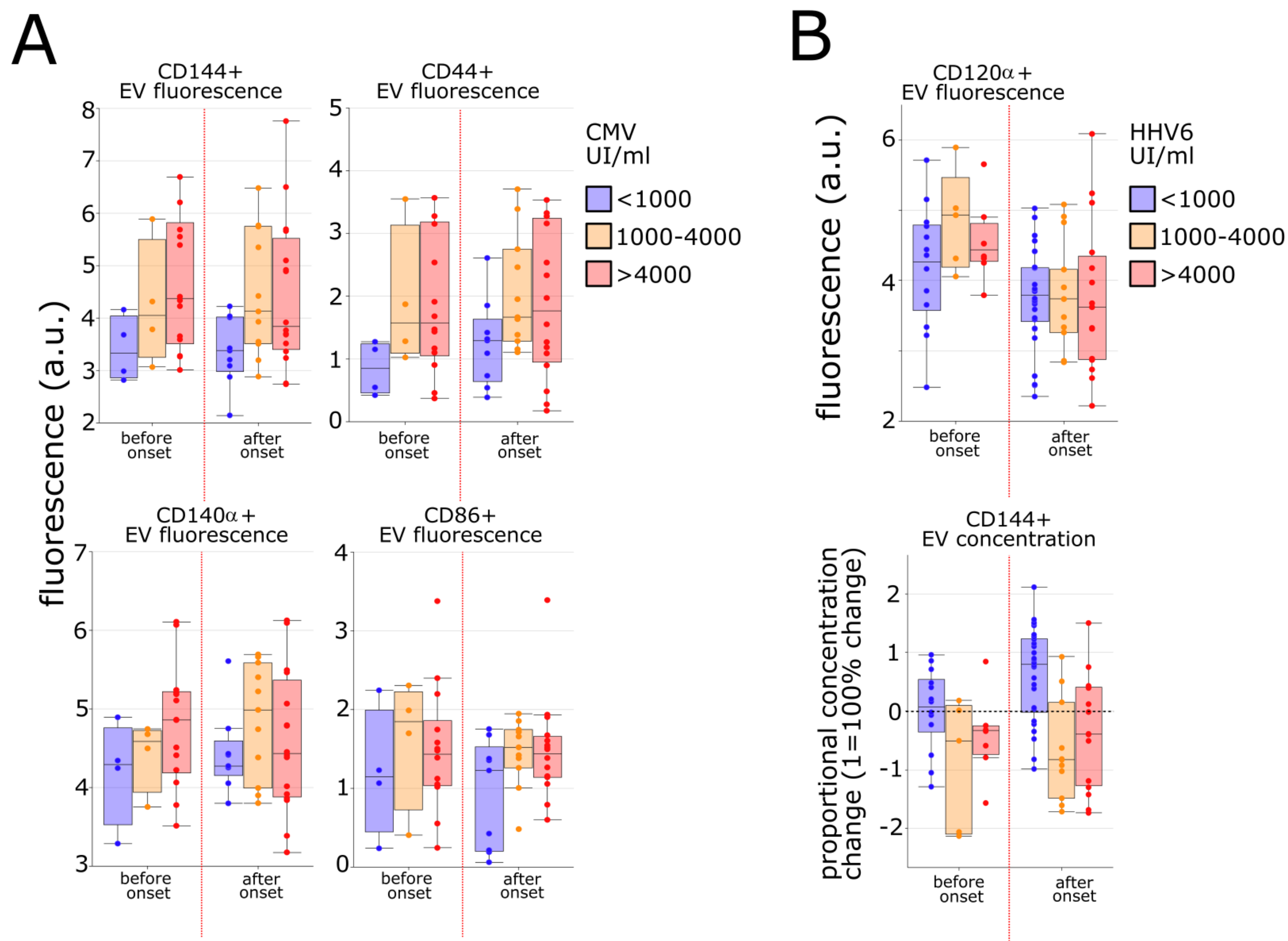

1.2 Supplemantary Tables

Table S1. Antibodies used in Guava experiments.

Antigen	Company	Clone	Alternative Name	Ligand/ Receptor
**Isotype FITC**	Miltenyi Biotech	REA293		
**Isotype PE**	Miltenyi Biotech	REA293		
**CD8**	Miltenyi Biotech	BW135/80	T8, Leu-2	MHC class I
**CD9**	Miltenyi Biotech	REA1071	5H9, MRP-1, TSPAN-29, p24, MIC3, Tspan29, ORF, GIG2	
**CD25**	BioLegend	BC96	Tac, p55, IL-2Ra	IL-2
**CD26**	Miltenyi Biotech	FR10-11G9	Dipeptidyl peptidase-4	
**CD30**	Miltenyi Biotech	KI-2	Ki-1, Ber-H2	CD153
**CD31**	Miltenyi Biotech	REA730	PECAM-1, endocam, GPIIa	CD138
**CD44**	Miltenyi Biotech	REA690	H-CAM, Pgp-1, Hermes antigen, ECMRIII	Hyaluronin, osteopontin, fibronectin
**CD81**	Miltenyi Biotech	REA513	CVID6, S5.7, TAPA1, TSPAN28	
**CD86**	Miltenyi Biotech	REA968	B70, B7-2	CD28, CD152
**CD106**	Miltenyi Biotech	REA269	VCAM-1, INCAM-110	CD49d/CD29, CD49d/β7
**CD120α**	Miltenyi Biotech	REA252	TNFR-1, TNFRSF1A	TNFa, TNFα
**CD138**	BioLegend	MI15	Syndecan-1	Extra Cellular Matrix
**CD140α**	BioLegend	16A1	PDGFRA, PDGFRa	PDGF-A, PDGF-B, PDGF-C
**CD144**	R&D Systems	123413	VE-Cadherin, Cadherin-5	B-Catenin
**CD146**	Miltenyi Biotech	541-10B2	MUC18, S-endo, MCAM, Mel-CAM	
**KRT18**	Abnova	C-04	Keratin 18 Type I, Cytokeratin-18, CYK18	C-Cbl, TRADD, Collagen, type XVII, alpha 1, DNAJB6, Pinin

Table S2. Univariate association analysis between EV surface biomarkers and CMV reactivation considering different time periods post-hHCT.

Marker analysis was performed using logistic regression model over 7-day intervals, considering either all time points post-hHCT or starting from 7 or 14 days post-hHCT (using a time-varying Cox proportional hazards model). The monotonic association between EV surface biomarker levels and CMV copies/mL was assessed via Spearman correlation analysis. ORs and HRs with p ≤ 0.2 are highlighted in grey. Spearman's rho values with p ≤ 0.05 are highlighted in grey, while those with p ≤ 0.1 are underlined.

Marker	*Type*	**Logistic Regression Model**												**Cox regression model**				**Spearman correlation**	
		Post-hHCT				*From +7d post-hHCT*				*From +14d post-hHCT*									
		change		absolute		change		absolute		change		absolute		change		absolute			
		OR	p	OR	p	OR	p	OR	p	OR	p	OR	p	HR	p	HR	p	rho	p
Total EVs	Conc	.842	.415	1.017	.92	.86	.491	1.131	.551	.829	.421	1.137	.579	.808	.184	.696	.19	.046	.870
CD8	MFI	.787	.239	1.077	.488	.805	.31	.977	.868	.82	.38	.884	.542	.856	.211	1.212	.399	.111	.695
	Conc	.763	.152	1.047	.711	.781	.171	.96	.753	.77	.198	.835	.357	.942	.708	.789	.33	-.232	.405
CD25	MFI	1.326	.158	1.015	.906	1.434	.081	1.052	.772	1.299	.194	1.146	.462	.832	.262	1.213	.487	.061	.830
	Conc	.911	.611	1	.997	.879	.504	.9	.47	.791	.212	.86	.353	.785	.163	.539	.037	-.018	.950
CD26	MFI	1.264	.207	1.142	.181	1.259	.214	1.156	.187	1.284	.163	1.316	.044	1.232	.187	1.146	.496	.243	.383
	Conc	.874	.519	1.046	.771	.891	.591	1.135	.532	.867	.532	1.155	.53	.829	.225	.662	.156	.161	.567
CD30	MFI	1.09	.671	1.031	.827	1.018	.937	.86	.407	.975	.917	.783	.299	1.304	.132	1.733	.054	-.071	.800
	Conc	1.361	.167	1.154	.296	1.308	.228	1.132	.43	1.228	.358	1.056	.736	1.205	.203	1.083	.72	.118	.676
CD31	MFI	.871	.479	1.05	.734	.909	.633	1.226	.317	.907	.625	1.215	.366	.937	.673	.919	.734	-.082	.771
	Conc	.905	.6	1.083	.572	.947	.779	1.247	.249	.951	.807	1.299	.278	.918	.612	.932	.806	-.061	.830
CD44	MFI	1.011	.95	1.003	.975	1.134	.482	1.349	.03	1.139	.459	1.349	.109	1.052	.77	1.068	.79	.289	.296
	Conc	1.184	.448	1.054	.721	1.253	.335	1.284	.259	1.277	.293	1.324	.236	1.044	.783	.887	.624	.150	.594
CD86	MFI	1.513	.094	1.609	.012	1.545	.102	1.958	.034	1.604	.088	2.337	.01	1.627	.002	2.486	.001	.607	.016
	Conc	1.12	.579	1.142	.236	1.121	.582	1.184	.334	1.131	.579	1.266	.314	1.036	.824	.908	.753	.593	.020
CD106	MFI	.971	.885	1.018	.891	.961	.846	1.018	.909	1.077	.73	1.283	.133	1.038	.805	.958	.837	.121	.666
	Conc	.996	.986	1.159	.436	1.015	.945	1.296	.294	1.03	.894	1.502	.168	.932	.619	.725	.285	.004	.990
CD120α	MFI	1.121	.651	1.085	.567	1.172	.554	1.229	.274	1.249	.431	1.512	.07	1.183	.249	1.44	.111	.354	.196
	Conc	.854	.445	1.019	.907	.87	.511	1.119	.601	.859	.495	1.186	.481	.889	.445	.826	.475	.054	.850
CD138	MFI	.869	.458	1.13	.395	.914	.649	1.288	.15	.913	.659	1.316	.191	1.232	.271	1.87	.016	.182	.516
	Conc	.892	.59	1.06	.722	.917	.692	1.215	.358	.877	.579	1.221	.401	.942	.712	.945	.826	.050	.860
CD140α	MFI	1.119	.582	1.145	.22	1.246	.291	1.445	.006	1.336	.183	1.871	.003	1.234	.141	1.415	.101	.482	.069
	Conc	.869	.501	1.036	.825	.888	.583	1.137	.541	.862	.514	1.141	.575	.84	.272	.764	.334	.096	.733
CD144	MFI	1.004	.984	1.204	.184	1.081	.682	1.571	.032	1.104	.618	1.846	.01	1.276	.088	2.232	.001	.743	.002
	Conc	.874	.512	1.062	.703	.9	.617	1.225	.337	.877	.558	1.255	.354	.913	.552	.897	.695	.114	.685
CD146	MFI	.985	.938	1.012	.924	1.002	.991	1.058	.732	1.042	.849	1.217	.317	.958	.778	.9	.667	.154	.585
	Conc	.919	.652	1.04	.789	.957	.818	1.187	.377	.939	.75	1.192	.428	1.003	.987	.954	.853	.061	.830
KRT18	MFI	.948	.797	1.235	.115	.965	.871	1.316	.135	.97	.893	1.358	.154	.981	.886	.944	.814	.025	.930
	Conc	.983	.936	1.094	.546	1.004	.984	1.17	.419	1.01	.963	1.214	.385	.944	.691	.757	.328	.014	.960

Abbreviations: MFI: Mean Fluorescence Intensity; Conc: Concentration of positive EVs (particles/ml of plasma); hHCT: Hematopoietic Stem Cells Transplantation; d: day; OR: odd ratio; HR: hazard ratio.

Supplementary Material

Table S3. **Univariate association analysis between EV surface biomarkers and EBV reactivation considering different time-periods post-hHCT.**

Marker analysis was performed using logistic regression model over 7-day intervals, considering either all time points post-hHCT or starting from 7 or 14 days post-hHCT (using a time-varying Cox proportional hazards model). The monotonic association between EV surface biomarker levels and EBV copies/mL was assessed via Spearman correlation analysis. ORs and HRs with p ≤ 0.2 are highlighted in grey. Spearman's rho values with p ≤ 0.05 are highlighted in grey, while those with p ≤ 0.1 are underlined

Marker	*Type*	**Logistic Regression Model**												**Cox regression model**				**Spearman correlation**	
		Post-hHCT				*From +7d post-hHCT*				*From +14d post-hHCT*									
		change		absolute		change		absolute		change		absolute		change		absolute			
		OR	p	OR	p	OR	p	OR	p	OR	p	OR	p	HR	p	HR	p	rho	p
Total EVs	Conc	1.028	.89	1.018	.901	.988	.953	.907	.588	1.045	.843	1.024	.91	1.105	.591	1.342	.403	.219	.518
CD8	MFI	1.05	.804	1.114	.327	1.018	.928	1.208	.159	.958	.84	1.459	.056	.909	.602	1.906	.128	.173	.611
	Conc	1.027	.894	1.041	.736	1.035	.868	1.013	.921	1.091	.711	.843	.358	.921	.679	1.171	.611	-.187	.582
CD25	MFI	.778	.238	1.212	.113	.769	.224	1.177	.333	.75	.182	1.358	.1	.684	.066	.892	.723	.319	.339
	Conc	1.018	.922	1.159	.129	1.048	.799	1.055	.673	1.085	.663	1.073	.648	.703	.115	1.068	.829	.469	.145
CD26	MFI	.808	.263	.934	.431	.798	.245	.861	.156	.804	.258	.8	.129	.868	.453	.83	.467	-.132	.699
	Conc	.942	.768	.986	.916	.894	.6	.856	.376	.939	.782	.957	.835	1.066	.732	1.3	.443	.205	.545
CD30	MFI	.757	.187	.833	.187	.765	.22	.865	.419	.743	.21	.828	.405	.58	.036	.546	.182	-.246	.466
	Conc	.592	.019	.917	.477	.573	.007	.854	.284	.613	.015	.947	.716	.702	.143	.895	.741	.005	.989
CD31	MFI	.664	.088	.784	.074	.616	.065	.605	.01	.667	.106	.681	.07	.776	.228	.811	.535	.128	.709
	Conc	.868	.511	.961	.766	.845	.449	.872	.423	.921	.723	1.012	.954	1.058	.798	1.678	.164	.132	.699
CD44	MFI	1.108	.574	1.028	.796	1.106	.606	1.001	.996	1.202	.352	1.148	.45	1.281	.273	1.223	.533	.005	.989
	Conc	.892	.611	1.02	.882	.856	.51	.908	.614	.918	.708	1.001	.998	1.043	.865	1.501	.291	.191	.573
CD86	MFI	.979	.921	1.013	.944	.963	.865	.902	.706	1.025	.911	1.008	.978	.846	.408	.372	.071	.005	.989
	Conc	.979	.921	1.162	.21	.954	.828	1.05	.764	1.052	.822	1.258	.288	1.088	.715	1.748	.189	.178	.601
CD106	MFI	.876	.552	.974	.837	.922	.714	1.082	.575	.935	.762	1.125	.488	1.013	.954	.907	.744	-.169	.620
	Conc	.864	.516	1	.999	.855	.483	.936	.741	.904	.665	1.108	.656	1.018	.922	1.17	.641	.100	.769
CD120α	MFI	.999	.997	.952	.682	.956	.835	.834	.232	.935	.763	.788	.193	.853	.468	.661	.259	.137	.689
	Conc	.899	.621	.974	.858	.853	.475	.827	.315	.874	.563	.886	.579	1.019	.916	1.162	.653	.278	.408
CD138	MFI	1.048	.817	1.027	.837	1.05	.814	1.02	.886	1.111	.636	1.179	.388	1.25	.423	1.129	.769	.123	.689
	Conc	.983	.93	1.074	.626	.942	.779	.963	.838	.997	.991	1.11	.617	1.105	.61	1.605	.251	.232	.492
CD140α	MFI	1.116	.548	1.027	.786	1.077	.704	.886	.361	1.023	.913	.771	.137	1.022	.907	.894	.737	-.355	.284
	Conc	1.003	.99	1.02	.891	.954	.822	.89	.511	1.003	.991	.993	.971	1.109	.597	1.51	.291	.150	.659
CD144	MFI	.944	.774	.898	.415	.933	.734	.829	.318	.991	.968	.951	.833	.869	.524	.639	.254	-.178	.601
	Conc	.989	.955	1.017	.907	.958	.839	.917	.64	1.032	.89	1.094	.685	1.131	.538	1.646	.217	.132	.699
CD146	MFI	.665	.093	.826	.087	.621	.071	.725	.034	.621	.07	.758	.157	.798	.243	.766	.504	.196	.564
	Conc	.781	.258	.993	.962	.761	.218	.865	.403	.802	.319	.92	.667	1.006	.978	1.429	.312	.415	.205
KRT18	MFI	1.133	.526	1.103	.441	1.228	.334	1.366	.075	1.236	.345	1.336	.166	1.412	.064	1.986	.043	.201	.555
	Conc	.965	.855	1.11	.434	.992	.97	1.17	.362	1.032	.883	1.27	.257	1.278	.197	2.193	.018	.324	.332

Abbreviations: MFI: Mean Fluorescence Intensity; Conc: Concentration of positive EVs (particles/ml of plasma); hHCT: Hematopoietic Stem Cells Transplantation; d: day; OR: odd ratio; HR: hazard ratio.

Table S4. Univariate association analysis between EV surface biomarkers and HHV6 reactivation considering different time-period post-hHCT.

Marker analysis was performed using logistic regression model over 7-day intervals, considering either all time points post-hHCT or starting from 7 or 14 days post-hHCT (using a time-varying Cox proportional hazards model). The monotonic association between EV surface biomarker levels and HHV6 copies/mL was assessed via Spearman correlation analysis. ORs and HRs with p ≤ 0.2 are highlighted in grey. Spearman's rho values with p ≤ 0.05 are highlighted in grey, while those with p ≤ 0.1 are underlined.

Marker	*Type*	**Logistic Regression Model**												**Cox regression model**				**Spearman correlation**	
		Post-hHCT				*From +7d post-hHCT*				*From +14d post-hHCT*									
		change		absolute		change		absolute		change		absolute		change		absolute			
		OR	p	OR	p	OR	p	OR	p	OR	p	OR	p	HR	p	HR	p	rho	p
Total EVs	Conc	.724	.107	.839	.203	.702	.084	.825	.263	.668	.079	.813	.322	.928	.687	1.158	.629	-.513	.030
CD8	MFI	.836	.355	.981	.864	.83	.36	.972	.841	.848	.447	.809	.32	.84	.264	1.536	.116	-.092	.716
	Conc	1.011	.955	.909	.431	1.024	.904	.895	.406	.917	.7	.903	.582	1.01	.958	.982	.95	.186	.461
CD25	MFI	1.224	.343	.853	.209	1.233	.324	.855	.349	1.239	.315	.889	.525	1.246	.235	1.14	.672	.021	.935
	Conc	.939	.734	.882	.181	.975	.895	.9	.417	.956	.822	.946	.729	.939	.786	.825	.598	.339	.169
CD26	MFI	1.044	.821	1.164	.092	1.016	.935	1.148	.205	.975	.894	1.101	.5	1.035	.851	1.06	.82	-.477	.046
	Conc	.764	.176	.854	.219	.755	.166	.864	.381	.731	.163	.864	.481	.941	.737	1.105	.755	-.526	.025
CD30	MFI	1.418	.097	1.151	.304	1.425	.115	1.107	.562	1.563	.082	1.295	.259	1.459	.078	1.505	.163	.359	.144
	Conc	1.261	.286	1.065	.594	1.258	.286	1.075	.609	1.265	.275	1.073	.625	1.135	.487	1.171	.563	-.542	.020
CD31	MFI	.991	.962	1.051	.711	.992	.967	1.075	.701	.968	.874	1.026	.9	.914	.568	.794	.411	.296	.232
	Conc	.738	.128	.869	.253	.726	.11	.86	.349	.707	.106	.854	.438	.956	.809	1.325	.377	-.463	.053
CD44	MFI	.849	.411	.994	.953	.824	.359	.946	.702	.813	.325	.872	.466	1.015	.933	1.095	.722	.555	.017
	Conc	.769	.166	.865	.239	.786	.218	.93	.69	.783	.227	.93	.716	1.011	.952	1.304	.327	-.169	.503
CD86	MFI	.884	.55	.674	.035	.908	.649	.764	.318	.918	.689	.781	.363	.918	.592	.887	.697	.391	.109
	Conc	.751	.15	.736	.009	.734	.136	.713	.047	.707	.139	.644	.058	.847	.366	.772	.505	.026	.919
CD106	MFI	1.082	.697	1.049	.697	1.077	.713	1.029	.832	1.049	.822	.973	.871	1.072	.699	1.088	.757	-.547	.019
	Conc	.809	.304	.852	.301	.805	.308	.844	.383	.773	.26	.809	.368	.899	.555	1.212	.556	-.657	.003
CD120α	MFI	.975	.9	1.106	.378	1.075	.721	1.365	.031	1.099	.659	1.424	.048	1.308	.144	2.257	.006	-.168	.506
	Conc	.766	.206	.879	.357	.776	.245	.932	.696	.745	.217	.937	.762	.99	.955	1.349	.291	-.502	.034
CD138	MFI	.94	.764	1.007	.954	.963	.86	1.066	.651	.9	.641	.922	.683	1.159	.425	1.629	.089	.234	.350
	Conc	.783	.227	.857	.27	.769	.213	.87	.428	.701	.134	.807	.303	.966	.854	1.216	.521	-.477	.046
CD140α	MFI	.883	.504	1.001	.994	.93	.714	1.131	.351	.945	.787	1.249	.202	1.108	.569	1.634	.053	-.333	.177
	Conc	.722	.116	.826	.151	.712	.104	.834	.281	.682	.1	.821	.337	.923	.672	1.203	.549	-.518	.028
CD144	MFI	.914	.647	.98	.878	.919	.674	.969	.867	.944	.792	1.048	.842	.907	.603	1.184	.508	-.211	.395
	Conc	.684	.055	.804	.111	.658	.037	.79	.184	.613	.033	.765	.235	.886	.5	1.182	.581	-.536	.022
CD146	MFI	1.36	.17	1.192	.124	1.438	.128	1.288	.099	1.538	.099	1.362	.111	1.203	.296	1.181	.563	-.397	.103
	Conc	.738	.128	.883	.342	.747	.15	.91	.563	.725	.139	.914	.629	.957	.806	1.302	.335	-.283	.255
KRT18	MFI	.962	.844	.923	.53	.936	.753	.86	.364	.92	.705	.822	.347	1.118	.502	1.355	.243	-.340	.168
	Conc	.811	.27	.861	.258	.786	.218	.817	.229	.734	.144	.736	.149	.978	.905	1.257	.398	-.635	.005

Abbreviations: MFI: Mean Fluorescence Intensity; Conc: Concentration of positive EVs (particles/ml of plasma); hHCT: Hematopoietic Stem Cells Transplantation; d: day; OR: odd ratio; HR: hazard ratio.

Table S5. Viral load effect. Univariate association analysis using logistic regression analysis between extracellular vesicle (EV) surface biomarkers and the effect of viral load (categorized as ≥1000 UI/mL, 1000–4000 UI/mL, and >4000 UI/mL) on reactivation, evaluated across different time periods post-hHCT (overall, ≥+7 days, and ≥+14 days) for CMV, HHV-6, and EBV. ORs with $p \leq 0.05$ are highlighted in grey, while those with $p \leq 0.1$ are underlined.

CMV	CMV>1000 UI/ml		CMV 1000-4000 UI/ml		CMV >4000 UI/ml	
**CMV post-hHCT**						
	OR	p	OR	p	OR	p
CD44 MFI	**1.003**	**0.975**	**1.083**	**0.579**	0.969	0.784
CD86 MFI	1.609	0.012	1.560	0.03	1.630	0.019
CD140α MFI	**1.145**	**0.220**	1.139	0.378	**1.148**	**0.229**
CD144 MFI	**1.204**	**0.184**	1.356	0.122	**1.144**	**0.350**
**CMV From +7d post-hHCT**						
	OR	p	OR	p	OR	p
CD44 MFI	**1.349**	**0.030**	***1.438***	***0.089***	*1.309*	*0.058*
CD86 MFI	**1.958**	**0.034**	1.709	0.182	**2.088**	**0.021**
CD140α MFI	**1.445**	**0.006**	***1.358***	***0.064***	**1.486**	**0.008**
CD144 MFI	**1.571**	**0.032**	1.484	0.167	**1.612**	**0.029**
**CMV From +14d post-hHCT**						
	OR	p	OR	p	OR	p
CD44 MFI	1.349	0.109	1.361	0.283	1.343	0.121
CD86 MFI	**2.337**	**0.010**	**2.170**	**0.032**	**2.276**	**0.014**
CD140α MFI	**1.871**	**0.003**	**1.723**	**0.020**	**1.950**	**0.004**
CD144 MFI	**1.846**	**0.010**	1.741	0.160	**1.901**	**0.005**

HHV6	HHV6>1000 UI/ml		HHV6 1000-4000 UI/ml		HHV6 >4000 UI/ml	
**HHV6 post-hHCT**						
	OR	p	OR	p	OR	p
CD120α MFI	**1.106**	**0.378**	**1.184**	**0.254**	1.030	0.826
CD86 MFI	**.674**	**0.035**	1.077	0.685	**.597**	**0.007**
CD144 Δconc	**.683**	**0.055**	**.460**	**0.009**	***.668***	***0.084***
**HHV6 From +7d post-hHCT**						
	OR	p	OR	p	OR	p
CD120α MFI	**1.365**	**0.031**	**1.592**	**0.011**	1.109	0.502
CD86 MFI	.764	0.318	1.293	0.313	.617	0.101
CD144 Δconc	**.658**	**0.037**	**.427**	**0.006**	***.657***	***0.087***
**HHV6 From +14d post-hHCT**						
	OR	p	OR	p	OR	p
CD120α MFI	**1.424**	**0.048**	**1.648**	**0.006**	1.155	0.518
CD86 MFI	.781	0.363	1.181	0.554	.654	0.148
CD144 Δconc	**.613**	**0.033**	**.368**	**0.001**	***.617***	***0.093***

EBV	EBV>1000 UI/ml		EBV 1000-4000 UI/ml		EBV >4000 UI/ml	
**EBV post-hHCT**						
	OR	p	OR	p	OR	p
CD30 Δconc	**.592**	**0.019**	**.866**	**0.737**	.678	0.254
KRT18 MFI	1.103	0.441	.966	0.914	1.058	0.701
**EBV From +7d post-hHCT**						
	OR	p	OR	p	OR	p
CD30 Δconc	.573	0.007	0.899	0.803	0.686	0.207
KRT18 MFI	1.366	0.075	1.074	0.866	1.287	0.218
**EBV From +14d post-hHCT**						
	OR	p	OR	p	OR	p
CD30 Δconc	.613	0.015	.913	0.829	.767	0.362
KRT18 MFI	1.336	0.166	1.075	0.834	1.143	0.580

Abbreviations: MFI: Mean Fluorescence Intensity; Conc: Concentration of positive EVs (particles/ml of plasma); Δ: change from basal pre-transplantation; hHCT: Hematopoietic Stem Cells Transplantation; d: day; OR: odd ratio; UI: internation unit.
